# Optimizing Phylogenomics with Rapidly Evolving Long Exons: Comparison with Anchored Hybrid Enrichment and Ultraconserved Elements

**DOI:** 10.1093/molbev/msz263

**Published:** 2019-11-09

**Authors:** Benjamin R Karin, Tony Gamble, Todd R Jackman

**Affiliations:** 1 Department of Biology, Villanova University, Villanova, PA; 2 Museum of Vertebrate Zoology and Department of Integrative Biology, University of California, Berkeley, CA; 3 Department of Biological Sciences, Marquette University, Milwaukee, WI; 4 Milwaukee Public Museum, Milwaukee, WI; 5 Bell Museum of Natural History, University of Minnesota, St. Paul, MN

**Keywords:** marker selection, reduced representation, sequence capture, anchored hybrid enrichment, ultraconserved elements, phylogenetics

## Abstract

Marker selection has emerged as an important component of phylogenomic study design due to rising concerns of the effects of gene tree estimation error, model misspecification, and data-type differences. Researchers must balance various trade-offs associated with locus length and evolutionary rate among other factors. The most commonly used reduced representation data sets for phylogenomics are ultraconserved elements (UCEs) and Anchored Hybrid Enrichment (AHE). Here, we introduce Rapidly Evolving Long Exon Capture (RELEC), a new set of loci that targets single exons that are both rapidly evolving (evolutionary rate faster than *RAG1*) and relatively long in length (>1,500 bp), while at the same time avoiding paralogy issues across amniotes. We compare the RELEC data set to UCEs and AHE in squamate reptiles by aligning and analyzing orthologous sequences from 17 squamate genomes, composed of 10 snakes and 7 lizards. The RELEC data set (179 loci) outperforms AHE and UCEs by maximizing per-locus genetic variation while maintaining presence and orthology across a range of evolutionary scales. RELEC markers show higher phylogenetic informativeness than UCE and AHE loci, and RELEC gene trees show greater similarity to the species tree than AHE or UCE gene trees. Furthermore, with fewer loci, RELEC remains computationally tractable for full Bayesian coalescent species tree analyses. We contrast RELEC to and discuss important aspects of comparable methods, and demonstrate how RELEC may be the most effective set of loci for resolving difficult nodes and rapid radiations. We provide several resources for capturing or extracting RELEC loci from other amniote groups.

## Introduction

Though large phylogenomic data sets have become relatively easy to obtain in recent years and have led to many highly resolved phylogenetic estimates, it has become clear that the sheer quantity of sequence data that can now be gathered will not unambiguously resolve some of the most difficult nodes in the tree of life. These difficulties may be caused by a number of factors including systematic error from nonphylogenetic signal or model inadequacy (Hahn and Nakhleh 2016; Reddy et al. 2017), gene tree estimation error from insufficient phylogenetic signal (Blom et al. 2017), or from natural processes such as incomplete lineage sorting and introgression (Maddison 1997; Edwards 2009) and positive selection (Castoe et al. 2009). Even as whole genomes have become easier to sequence for phylogenomics, they must still be subsetted to make aligned sets of orthologous loci. Appropriate marker selection is therefore a critical part of phylogenomics, and it is still under considerable debate what kinds of markers are the best for resolving difficult branches at various evolutionary depths. For example, questions remain whether to use coding or noncoding sequence data (Chen et al. 2017; Reddy et al. 2017), conserved or highly variable loci (Salichos and Rokas 2013; Betancur-R et al. 2014), long or short alignments (Edwards et al. 2016; Springer and Gatesy 2016), single nucleotide polymorphisms alone (SNP) or full sequence alignments (Leaché and Oaks 2017), or other types of markers that may be relatively free of homoplasy (e.g., indels, Simmons and Ochoterena 2000; SINEs, Ray 2006; transposable elements, Han et al. 2011; micro RNAs, Tarver et al. 2013). Each kind of marker possesses different trade-offs of phylogenetic information content (PIC), maintenance across evolutionary scales, susceptibility to error, and computational tractability, and these factors must be balanced during marker selection. A universal marker type for all kinds of phylogenetic questions is unlikely to exist, which may necessitate filtering for question-specific markers (Chen et al. 2015) as just a small number of loci out of thousands may have the power to resolve specific questions or drive a contentious pattern (Brown and Thomson 2017; Shen et al. 2017). Therefore, careful marker selection before sequencing to prioritize signal and resolving power over sheer quantity of data may be an important step forward in phylogenomics.

### Marker Selection Trade-Offs

A main trade-off in marker selection is to maximize per-locus phylogenetic signal without suffering alignment quality or excessive substitution saturation. In selecting markers, PIC can be improved either by increasing the length of the locus or by choosing loci with a faster rate of evolution. Both of these strategies increase the number of phylogenetically informative sites that can increase phylogenetic resolution (Graybeal 1994). Given sufficient time, substitution saturation will eventually erode phylogenetic signal in all types of markers (Graybeal 1994), but this will occur fastest on loci that evolve more rapidly (Yang 1998). At shallow evolutionary scales it is generally agreed that rapidly evolving markers are the most effective for resolving trees because substitution saturation is unlikely to have occurred and variable markers are more likely to contain phylogenetic signal for relationships with short internodes (Dornburg et al. 2017). On the other hand, it is still under considerable debate whether conserved or variable markers are more effective at resolving deeper relationships (Philippe et al. 2011; Salichos and Rokas 2013; Betancur-R et al. 2014), as one must balance the depth of the relationships, the rate of character change affecting the chance that the signal will be masked, and the internode length since conserved loci may not have undergone any substitutions along a short branch (Townsend et al. 2012). A marker with a particular rate can have high phylogenetic utility when internodes are long, but be positively misleading when short (Dornburg et al. 2017).

Choosing longer loci will also lead to increased PIC, but one must balance another trade-off: short loci are more likely to suffer from gene tree estimation error (GTEE) due to low signal-to-noise ratio (Betancur-R et al. 2014) while long loci are more likely to carry past recombination events with different parts holding different genealogical histories (Degnan and Rosenberg 2009), and both issues are statistically inconsistent under the multispecies coalescent (Arcila et al. 2017). GTEE has emerged as a major potential pitfall in phylogenomics with summary coalescent species tree methods being particularly susceptible (Gatesy and Springer 2014; Mirarab, Bayzid, et al. 2014; Roch and Warnow 2015). Summary coalescent species tree methods are the most commonly used application of the multispecies coalescent to phylogenomic data sets due to computational limitations for conducting full Bayesian coalescent approaches (Mirarab, Reaz, et al. 2014). The negative implications of GTEE on summary methods has prompted the implementation of tactics such as binning alignments based on information content (which involves concatenating alignments that return similar topologies in the hope of obtaining more accurate “gene tree” estimates; Mirarab, Bayzid, et al. 2014), or limiting data sets to high-resolution genes (Chen et al. 2015). Some methods that interpret gene trees based on quartet subtrees may be more robust to GTEE when large amounts of data (millions of SNPs or several thousands of loci) are provided, such as ASTRAL (Mirarab and Warnow 2015) or SVDquartets (Chifman and Kubatko 2014) though they should be tested more rigorously (Roch and Warnow 2015). It may be preferable to initially select markers that hold high PIC in order to directly reduce per-locus GTEE instead of post hoc methods to reduce it. As computational resources continue to improve, future studies may be able to use preferable full Bayesian coalescent species tree methods if they instead target a reduced number of particularly informative loci (Ogilvie et al. 2016). For example, StarBEAST2 (Ogilvie et al. 2017) is faster than previous methods such as *BEAST (Ogilvie et al. 2016; Posada 2016) and these methods have both been used either by subsetting loci (Blom et al. 2017) or tips (Bragg et al. 2018). These factors indicate the necessity for a shift in the current paradigm in phylogenomic data acquisition and analysis. Future phylogenomic studies should focus on sequencing fewer loci that are longer and provide greater phylogenetic signal and optimal evolutionary rates to answer specific questions (Leaché et al. 2016). These kinds of markers are the least likely to suffer from GTEE and will also provide higher resolution for estimating the species tree (Salichos and Rokas 2013; Mirarab, Bayzid, et al. 2014; Roch and Warnow 2015).

Another important consideration in marker selection is the difference between protein-coding and noncoding sequences, as in some systems each data type has produced quite different phylogenetic estimates (Lavoué et al. 2003; Nikolaev et al. 2007; Shaw et al. 2007; Reddy et al. 2017). Natural selection on noncoding regions can range from highly purifying (e.g., ultraconserved elements, Katzman et al. 2007) to relatively neutral (e.g., introns, Prychitko and Moore 1997; Chamary et al. 2006). Of markers that can be confidently determined to be orthologous, introns may have the fastest rate of evolution and have been used with success to produce well-resolved phylogenies (Jarvis et al. 2014; Chen et al. 2017), yet alignments can be problematic for distantly related taxa as evolution is relatively unconstrained (Li et al. 2017). Coding sequences also evolve under a broad range of selective regimes, but are likely to always undergo some purifying selection on the resulting protein structure (Graur and Li 2000). For example, frame-shifts from indels causing premature stop codons are likely to be highly deleterious. Furthermore, codon positions can evolve at substantially different rates due to their propensity for synonymous versus nonsynonymous mutations, though substitutions even in more neutrally evolving third codon positions may still be biased. In the avian phylogeny, initial concerns about the utility of coding data were that genomic-scale molecular convergence in coding sequences due to GC-biased gene conversion or other means may bias phylogenetic estimates (Jarvis et al. 2014). Instead, it is most likely that model inadequacy directly associated with at least one of the data types explains the different estimates (Reddy et al. 2017). Still, more complex models of nucleotide evolution that incorporate additional parameters for such observed differences as individual codon substitution rates (Yang and Nielsen 2002), corresponding protein structures (Whelan and Goldman 2001), or biased conversion at synonymous sites (Galtier and Gouy 1998; Holland et al. 2013) are likely to improve model fit and phylogenetic estimates for coding sequences (Dornburg et al. 2017).

### Phylogenomic Data Set Types

Several targeted hybrid enrichment data sets have been developed for phylogenomics (Faircloth et al. 2012; Lemmon et al. 2012; Singhal et al. 2017) that enable researchers to capture the same sets of markers across all taxa of interest and exclude repetitive or otherwise phylogenetically misleading parts of the genome (such as pseudogenes and paralogs). The benefit of consistently using the same sets of markers across studies is that it will eventually allow for meta-analyses as more data accumulates (Lemmon et al. 2012; Streicher and Wiens 2017). Within amniotes, the most commonly used reduced representation data sets for phylogenomics are ultraconserved elements (UCEs, Faircloth et al. 2012) and Anchored Hybrid Enrichment (AHE, Lemmon et al. 2012), both of which were developed to target the variable flanking regions surrounding highly conserved anchor points, and we describe these in more detail below. Conserved nonexonic elements (CNEEs; Edwards et al. 2017) are another recently proposed reduced representation data set, though these loci may suffer from gene trees with low per-locus bootstrap support when compared with UCEs and introns and their utility has yet to be rigorously tested. Transcriptome data itself can be used for phylogenomics (Figuet et al. 2014; Wickett et al. 2014; Brandley et al. 2015), however, in order for data sets to be consistent the transcriptomes must be gathered from the same tissue types and when analyzing transcriptome data it can be difficult to accurately assess orthologs and align different isoforms. Finally, exon capture is commonly used to sequence orthologous exons across taxa for phylogenomics, though the loci are usually not consistent across studies, as researchers usually build unique probes for their focal group. In nonmodel organisms, exon capture probes are usually designed by first sequencing a transcriptome (TBEC; Bi et al. 2012), but it can be difficult to determine intron–exon boundaries for TBEC probe design, which in some cases could reduce capture efficiency (but see Portik et al. 2016).

UCEs are regions of the genome (≥100 bp in length) that have high sequence identity (≥80%) across extremely divergent taxa (Faircloth et al. 2012). UCEs are often in noncoding genomic regions, though some UCEs correspond to exons (Bejerano et al. 2004). A unifying functional role of UCEs is not fully understood (Harmston et al. 2013), though they have been shown to often play a role in gene regulation (Lenhard et al. 2003; Woolfe et al. 2004; Ni et al. 2007; Warnefors et al. 2016) and development (Dickel et al. 2018) and are undergoing purifying selection about three times higher than coding regions (Katzman et al. 2007). The UCE tetrapod probeset includes ∼5,000 loci, captured using 120 bp probes (Faircloth et al. 2012). The AHE vertebrate data set targets a much smaller number of loci compared with UCEs (400–500 depending on the study), similarly focusing on regions of the genome that are conserved across vertebrates (but not as high identity as UCEs) and flanked by more variable regions (see Lemmon et al. 2012 for additional criteria). Over 90% of AHE probe regions correspond to exons, however, the flanking regions include a higher proportion of introns or other genomic elements (see fig. 2 of Lemmon et al. 2012). In both of these sets, the majority of the phylogenetically informative sites are expected to exist in the flanking regions rather than the more conserved probe regions, in theory to allow for the most effective capture during hybridization. However, since its inception (Lemmon et al. 2012), AHE has shifted from this “anchor” method toward tiling probes across a substantially longer target region for a reduced number of loci (Prum et al. 2015; Ruane et al. 2015), highlighting the advances in sequence capture technology allowing for hybridization to highly diverged sequences (e.g., Li et al. 2013). When employed, both the UCE and AHE data sets have often been able to resolve previously difficult nodes (e.g., Crawford et al. 2012, 2015; Prum et al. 2015; Bryson et al. 2016; Streicher and Wiens 2017), though short length and/or or slow evolutionary rate may make both methods susceptible to GTEE as we show in this study.

### Rapidly Evolving Long Exon Capture

Here, we introduce a set of loci optimized for high-resolution phylogenomic inference: Rapidly Evolving Long Exon Capture (RELEC). We selected these loci to maximize PIC while maintaining presence, orthology, and alignment quality across broad evolutionary scales. RELEC loci may provide the most accurate phylogenetic resolution at deep and shallow divergences, while also remaining computationally tractable. While UCEs, and AHE in a similar but less extreme extent, were developed by applying a maximum evolutionary rate cutoff within a core probe region, our RELEC approach is unique in that we apply a minimum rate cutoff across long orthologous regions in order to maximize PIC and to produce robust gene trees.

Long and rapidly evolving genes hold abundant phylogenetic signal, thus increasing the chance of producing reliable individual gene trees and decreasing stochastic error associated with short genes (Salichos and Rokas 2013; Lanier et al. 2014; Mirarab, Bayzid, et al. 2014; Shen et al. 2016) while retaining many other benefits of exons that have led to their continued use in phylogenetics and phylogenomics. Benefits of exons include: 1) nucleotide evolution can be modeled more complexly than noncoding regions based upon observed rate differences between different kinds of substitutions. 2) Protein functions are often known, which in some cases may allow for studies of phenotypic or functional differences between organisms; 3) Aligning protein-coding regions is straightforward, and translation-based alignment algorithms are accurate (Abascal et al. 2010). No other kind of marker can be easily aligned at up to 40% sequence divergence. 4) Indels can be accurately aligned in exons because they occur predictably in multiples of 3 bp due to selection against reading frame shifts and can also be valuable phylogenetic characters on their own (Simmons and Ochoterena 2000; Luan et al. 2013).

We compared the utility of RELEC, AHE, and UCE loci within the squamate phylogeny by extracting the markers from 17 genomes spanning ∼200 Ma divergence. We also provide sequence data for the RELEC loci extracted from available mammal and bird genomes, making these loci easy to implement in phylogenomic studies in any amniote group (Supplementary Material online; https://github.com/benrkarin/RELEC). We find that the selected RELEC loci are long and highly informative and can still be accurately aligned at deep divergences, while at the same time avoiding the difficulties of orthology detection. No other set of loci for phylogenomics encompasses sequences that are as long and as variable without running into alignment problems at deeper evolutionary scales. This therefore sets up the RELEC loci to be a powerful tool for resolving recalcitrant nodes in the tree of life.

## Results and Discussion

### Assembling the RELEC Loci

By aligning and comparing exons across mammal, bird, and squamate genomes, we found 179 exons that fit the RELEC criteria (see Materials and Methods for more details). Likely due to their rapid evolutionary rate, many of the RELEC genes are poorly annotated in nonmammalian genomes, which required us to manually extract and compare each candidate locus using translated sequence TBlastN searches. By carefully assembling the data set in this way, we are highly confident that they all are both present among amniotes and will not present issues of paralogy that would lead to incorrect tree estimates. Still, not all lineages have the complete set of 179 long exons, often due to missing data in the genomes or in a few cases from deletions (see supplementary table S4, Supplementary Material online). For example, *ENAM* cannot be found in the chicken and painted turtle genomes, likely because these lineages do not have teeth and do not need the enamolin protein. *RIKEN* appears to be deleted in the human genome, but is present in mouse and other mammal genomes. We found only two cases of duplications within amniotes for the final set of RELEC loci, but we chose to retain them because they are highly informative and the paralogy histories are clear and easy to trace. The exceptions are the sperm receptor protein, *PKDREJ*, which shows three tandem duplicates within squamates, and *CKAP5L*, which was duplicated from *CKAP5* (which does not have a single long exon) near the reptile ancestor, as it is present in crocodiles, turtles, squamates, tuatara, and some birds, but is not present in mammals, *Xenopus*, coelacanth, gar, and zebrafish.

**Fig. 1.**
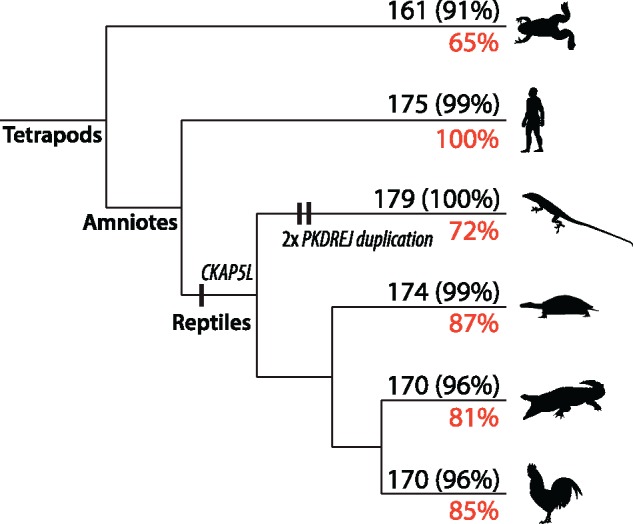
Tetrapod cladogram indicating the number of RELEC genes present in the model species for each of the main lineages, and in parentheses the percent of the total after accounting for the indicated duplication events. In red the percent of RELEC genes that are correctly annotated on Ensembl. Poor genome annotation outside of mammals is likely due to the rapid evolutionary rate of RELEC genes, as well as missing data in those genomes. Vertical dashes show the two exceptions where we allowed paralogs to be retained: two tandem duplications in *PKDREJ* in squamates and a duplication of *CKAP5* in reptiles. Most of the RELEC genes are present in *Xenopus*, though some are reduced in length, and many are likely present in higher vertebrate lineages as well. Silhouttes from http://phylopic.org, used under the Creative Commons (https://creativecommons.org/licenses/by/3.0/), with drawings of *Xenopus* and *Anolis* by Sarah Werning, and painted turtle by Scott Hartman.

After extracting the loci, we used TBlastN searches to Ensembl to confirm presence and proper annotation in other tetrapod lineages. In the human genome, all RELEC genes were correctly annotated, but nonmammalian genomes had much less accurate annotations (see fig. 1). The chicken, painted turtle, and saltwater crocodile genomes had 81–87% of RELC loci properly annotated, while *Anolis* had only 72%, and *Xenopus* only 65%. Annotation errors included incorrectly placed intron–exon boundaries, missing data, and no annotation at all. We found 161 loci in *Xenopus*, though 11 of these are reduced in size <1,400 bp, whereas in amniotes they are all retained >1,500 bp (with a few slight exceptions just below the cutoff; see supplementary table S4, Supplementary Material online). This indicates that a subset of the RELEC loci are retained at evolutionary scales beyond amniotes or tetrapods, and RELEC orthologs can likely be found in other groups as well.

### Gene Tree-Species Tree Discordance

**Fig. 2.**
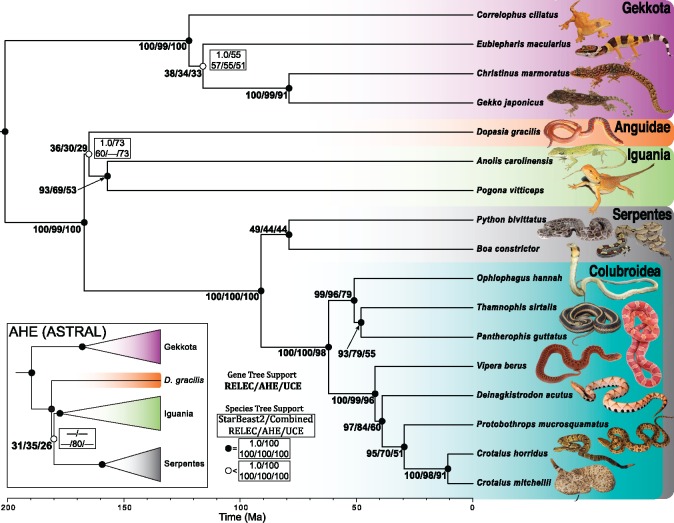
Time adjusted phylogenetic species tree for 17 squamate species using Rapidly Evolving Long Exon Capture (RELEC), Anchored Hybrid Enrichment (AHE), and Ultraconserved Element (UCE) data sets. The topology is the resulting ASTRAL species tree of the combined UCE, AHE, and RELEC data set, which matches the UCE and RELEC ASTRAL trees as well as the StarBEAST2 species tree for RELEC. Values to the left of the node represent the proportion of maximum likelihood gene trees that support a given node (RELEC/AHE/UCE). Circles on nodes correspond to support values from ASTRAL analyses; filled circles represent 100% support for all species tree analyses (including StarBEAST2) and open circles represent any node with than 100% in any of the data sets. Values to the right of the nodes give ASTRAL support values for branches with <100% support. The ASTRAL topology for AHE is inset, with support for the differential node shown.

**Fig. 3.**
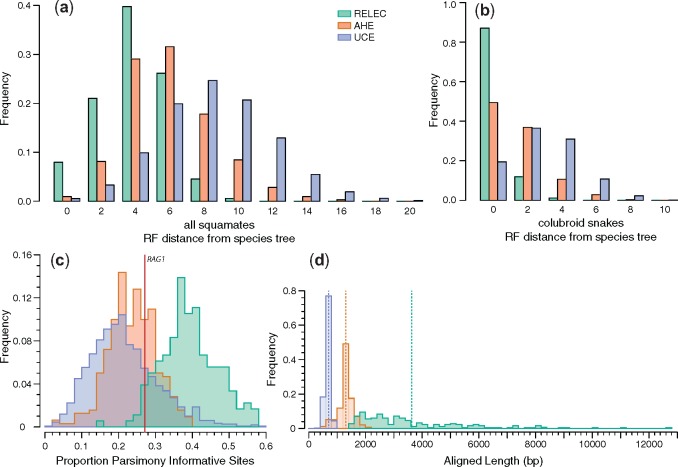
Histograms comparing features of RELEC, AHE, and UCE loci, with the *y* axis in each corresponding to the proportion out of 1. (*a* and *b*) Robinson–Foulds distances of maximum likelihood gene trees to full squamate species tree (combined RELEC, AHE, and UCE data set ASTRAL topology of fig. 1) and species tree limited to colubroid snakes. (*c*) Proportion per-locus of parsimony informative sites. (*d*) Comparison of aligned length of each locus in each data set, with dashed vertical lines indicating the mean length in each data set.

In this study, we estimated maximum likelihood (ML) gene trees and coalescent species trees using our newly designed RELEC data set, and independently using the AHE and UCE data sets, as well as combined analyses of all three data types (which we refer to as the “species tree”). All three data sets (RELEC: 179 loci, 651,434 bp; AHE: 320 loci, 427,251 bp; UCEs: 1,517 loci, 1,031,286 bp; see table 1) reconstruct the squamate phylogeny according to the species tree with minor differences at poorly supported nodes (fig. 2; Wiens et al. 2012; Pyron et al. 2013; Zheng and Wiens 2016; Streicher and Wiens 2017). Our comparison of sequence alignments for each set show that the RELEC loci as a whole are significantly longer and contain many more parsimony informative sites than both the UCE and AHE loci (fig. 3). The AHE alignments show significantly lower proportions as gaps compared with RELEC and UCEs (Mean ± SD; RELEC: 0.085 ± 0.076; AHE: 0.049 ± 0.081; UCEs: 0.076 ± 0.051; see supplementary fig. S6, Supplementary Material online, for other gap metrics).

**Table 1.** General Comparison of the Three Data Sets, As Acquired from the Squamate Genomes for This Study.

Data Set	Number of Markers	Mean Aligned Length (SD)	Range Aligned Length	Total Number Aligned Bases	Completeness (% w/17 Taxa)
RELEC	179	3,639 (2,015)	1,407–12,726	651,434	98
AHE	320	1,335 (453)	213–2,164	427,251	100
UCEs	1,517	680 (90)	179–985	1,031,286	100

We first assess accuracy by comparing the ASTRAL and concatenated species tree estimates of the squamate tree for each data set. All ASTRAL species tree nodes display strong 100% bootstrap (BS) support with two exceptions (also see open node circles in fig. 2). 1) Both RELEC and UCEs support *Dopasia gracilis* as sister to the iguanians, *Anolis carolinensis* and *Pogona vitticeps* (BS, RELEC: 60; UCEs: 73; Combined: 73) in agreement with the combined species tree, our RELEC StarBEAST2 estimate, and other published trees (e.g., Streicher and Wiens 2017). In contrast, both the ASTRAL and concatenated AHE trees recover support for *D. gracilis* sister to a clade composed of Iguania with snakes (BS, AHE: 80). Incomplete lineage sorting may explain the discordance on this short branch, as there are roughly equal 33% proportions for three quartet topologies at the node in each data set (see fig. 2), and low taxon sampling and insufficient phylogenetic signal are other possible explanations. Despite this, the RELEC StarBEAST2 analysis recovered strong (PP = 1.0) support for this placement (supplementary fig. S1, Supplementary Material online), suggesting a benefit to using full Bayesian methods to coestimate the gene trees and species tree together. 2) Within Gekkota, the ASTRAL trees for all three data sets recover the same topology, though there exists reduced support for the placement of *Eublepharis macularius* (BS, RELEC: 57; AHE: 55; UCEs: 51; Combined: 55) and the concatenated trees showed different placements of *E. macularius* (supplementary fig. S2, Supplementary Material online). The translated RELEC amino acid data ASTRAL and concatenated trees matched the species tree exactly and showed similar support to the RELEC nucleotide analyses (supplementary fig. S3, Supplementary Material online). Separate MP-EST (Liu et al. 2010) analyses on the same sets of gene trees showed nearly identical results to those presented here (results not shown).

**Fig. 4.**
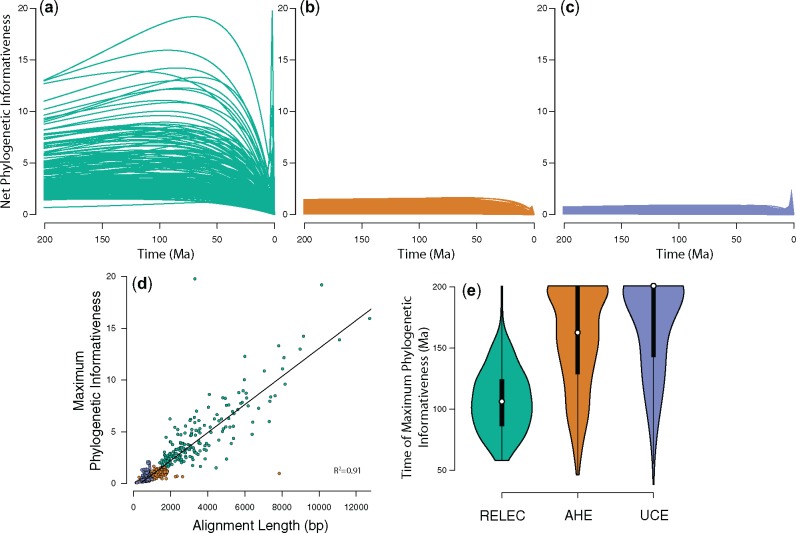
Phylogenetic informativeness of individual loci in the three data sets: (*a*) RELEC; (*b*) AHE; and (*c*) UCEs. The *y* axis is relative, and corresponds to the normalized, asymptotic likelihood that there will exist a mutation that accurately resolves a node at that point in time. The timescale corresponds to the same time-adjusted phylogeny in figure 1. (*d*) The significant relationship between alignment length and the maximum value of phylogenetic informativeness reached for each locus along the curve. (*e*) Violin plots of the time for which each locus reaches its maximum phylogenetic informativeness, with RELEC loci optimized to have high information content at significantly younger timescales than AHE and UCEs.

To assess the relative power of a gene to resolve a node at a given time period in the phylogeny, we generated phylogenetic informativeness profiles for each locus in each of the three data sets. Indeed, RELEC loci show considerably higher phylogenetic informativeness of each marker over the past 200 Ma (fig. 4). This is due in part to their length, which is significantly correlated with informativeness (fig. 4*d*), as well as their rate of evolution. RELEC loci also show a much higher proportion of parsimony informative sites than both UCEs and AHE (fig. 3*c*). The informativeness profiles also show that RELEC loci are most informative at resolving younger nodes compared with AHE and UCEs (see fig. 4*e*). Overall, our results show that the length and rate of RELEC loci provides them with extremely high phylogenetic informativeness across most relevant timescales and should therefore produce a greater proportion of individually reliable gene trees than do AHE and UCEs.

**Fig. 5.**
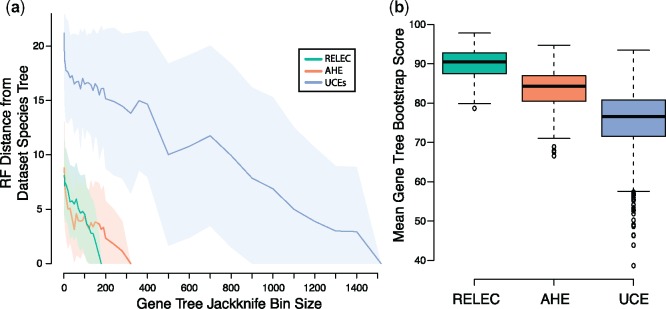
(*a*) RF Distance of gene trees of various bin size, divided between RELEC, AHE, and UCEs. ASTRAL analyses were run on gene tree bins of increasing size with 100 random jackknife replicates each, and then compared with the species tree estimated from the largest bin. The mean RF distance is depicted by the dark line, with 1 SD shown by the surrounding shaded area. (*b*) Boxplots of mean bootstrap scores for ML gene trees of each data set, with ANOVA and all comparisons significant by Tukey HSD test.

We quantified GTEE between data sets using average gene tree bootstrap scores, which provide a measure of gene tree confidence (Edwards et al. 2017), and by comparing each ML gene tree to the species tree. RELEC gene trees hold significantly higher average BS support (fig. 5*b*) and display higher per-node support than AHE and UCEs for all nodes in the tree that had <100% of the gene trees matching that node (fig. 2). Similar to the results of Singhal et al. (2017) who compared UCEs with AHE in squamates, we also find that AHE gene trees show higher average BS support and equal or higher per-node support than UCEs (figs. 2 and 5*b*). RELEC and AHE performed similarly when gene trees were divided into equal-sized bins of loci analyzed with ASTRAL, with many of the jackknife replicates matching the full (data set-specific) species tree with a bin size of ∼120 loci, whereas UCEs required 500–1,000 loci to reach a similar accuracy for the species tree estimate (fig. 5*a*). The robustness of individual RELEC gene trees was especially apparent in four uncontroversial nodes in the squamate tree where 93–97% of RELEC gene trees matched the species tree for that node while AHE and/or UCEs showed substantially lower proportional accuracy (AHE: 69–84%; UCEs: 51–60%); the *Anolis–Pogona* node, the *Thamnophis–Pantherophis* node, the *Protobothrops–Crotalus* node, and the *Deinagkistrodon–Protobothrops–Crotalus* node (see fig. 2). This suggests that RELEC gene trees are more likely to accurately resolve well-established nodes than AHE or UCEs, and this will likely apply to more difficult nodes as well. Furthermore, RELEC gene trees, on an average, showed lower RF distances to the species tree than AHE or UCEs (mean RF distance, RELEC: 4, AHE, 6, UCEs: 8), indicating not only higher per-node support but more gene trees matching the species tree in its entirety (fig. 3*a*).

We expect that RELEC markers will be most useful at resolving more recent divergences, so we also compared gene trees to the species tree for the colubroid snakes subclade, which is the most-sampled clade in our analysis (see fig. 2). Pyron et al. (2014) found substantial gene tree-species tree discordance among AHE loci for colubroid snakes (though these same difficult nodes are not present in this study). We find that RELEC gene trees show reduced discordance in colubroid snakes than AHE or UCE gene trees (percent of gene trees matching species tree, RELEC: 87%; AHE: 49%, UCEs: 19%; see fig. 3*b*). We therefore expect that RELEC loci may more accurately resolve this clade if applied to the group as a whole, and be powerful at resolving recent radiations in general.

When examining the entire squamate tree, <1% of UCE or AHE gene trees match the species tree exactly, while 8.0% of RELEC gene trees match it. Though given the poor-support for the placement of *D. gracilis* in most analyses and possible incomplete lineage sorting on this short branch, if we include gene trees that differ by one node (fig. 3b, RF = 2) then a much larger proportion of gene trees are accurate (RELEC: 28.8%; AHE: 9.0%; UCEs: 3.8%). Despite this low overall rate, all three methods stand in stark contrast to the results of studies with expansive genomic-scale data sets where none of the individual gene trees matches the species tree (Salichos and Rokas 2013; but see Betancur-R et al. 2014; Jarvis et al. 2014; Arcila et al. 2017). Higher discordance and GTEE in UCEs may be due to their shorter alignment lengths, lower phylogenetic signal, and less clocklike rates (Singhal et al. 2017), potentially leading to increased stochastic error for individual UCE gene trees compared with AHE or RELEC gene trees. Substitutions along short branches are less likely to have occurred for conserved loci, which may explain why relatively conserved AHE and UCE data sets have previously shown poor support for areas of the trees undergoing rapid diversification (McCormack et al. 2013; Pyron et al. 2014). As GTEE can have strong negative impacts on summary-coalescent species-tree analyses (Roch and Warnow 2015), RELEC loci that minimize it may also be the most effective for these tree-building methods.

### Utilizing Full Bayesian Coalescent Species Tree Methods

Though normally too computationally demanding to be utilized in phylogenomic-sized data sets, given the relatively small taxon-set in this study, we were able to carry out a Bayesian coalescent species tree analysis on the RELEC data set using StarBEAST2. We visualized results from both MCMC chains in Tracer 1.6 (Rambaut et al. 2018) and both runs reached stationarity relatively quickly, with total computation times between 70 and 75 h using 24 threads. The analysis converged on the same species tree as the combined data set ASTRAL species tree, with posterior probabilities of 1.0 for all nodes (supplementary fig. S1, Supplementary Material online). Furthermore, there appeared to be little species-tree discordance even at poorly supported nodes in ASTRAL analyses (fig. 2), indicating strong support for the sister relationship between Anguidae and Iguania. Given current computational power available to most researchers, we are unsure if it is possible to use StarBEAST2 for analyses of data sets much larger than this study as number of tips increases computation time rapidly (Ogilvie et al. 2016, 2017), however, our results show promise for the future possibility of using these comprehensive Bayesian coalescent species tree analyses for phylogenomic data sets with fewer but longer and highly informative loci.

### Substitution Saturation

**Fig. 6.**
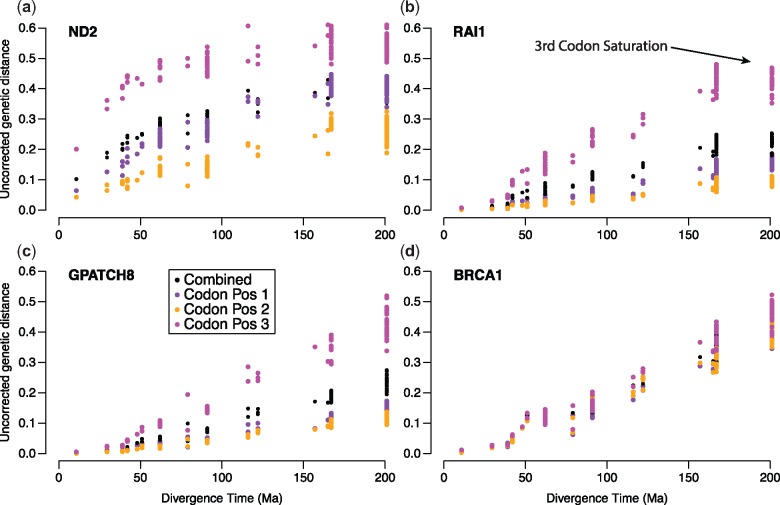
Plots to assess substitution saturation for three RELEC loci: (*b*) *RAI1*, (*c*) *GPATCH8*, and (*d*) *BRCA1* based on comparison of raw pairwise sequence distance versus divergence time. (*a*) Mitchondrial *ND2* is shown for reference. A signature of saturation is present where pairwise distance does not increase despite increasing divergence time, which is prevalent in all three codon positions in *ND2*. Most RELEC loci show a pattern similar to *GPATCH8* or *BRCA1*, with a linear increase in pairwise distance over time, while *RAI1* shows some evidence of third codon saturation after 160 Ma (see arrow). For all saturation plots, see supplementary figure S4, Supplementary Material online.

Substitution saturation decreases phylogenetic signal in sequence data by masking relevant mutations for phylogenetic inference, and has negatively affected the ability to reconstruct deep nodes in the tree of life. It is especially common in mitochondrial genes, as they evolve quickly and have a lower effective population size (Jackman et al. 1999). Researchers may be concerned that some RELEC markers may experience saturation at even moderate divergences because they evolve so rapidly, as substitution saturation has often been implicated as a problem for phylogenomics (Jeffroy et al. 2006; Parks et al. 2012; Breinholt and Kawahara 2013; Dornburg et al. 2014). However, saturation of nuclear genes normally only affects studies attempting to reconstruct deep relationships, such as the placement of turtles with respect to other reptiles (Chiari et al. 2012) and crown vertebrate lineages (Dornburg et al. 2014). RELEC loci should primarily be used to recover younger relationships within (not between) the major amniote groups, such as relationships among or within snake, gecko, or iguanian families. Within these timescales, saturation plots for each RELEC locus up to 200 Ma divergence times overall show little evidence of substitution saturation at moderate timescales (fig. 6 and supplementary fig. S4, Supplementary Material online). In just a few cases the third codon position appears to show a signature of saturation by 160 Ma (e.g., *RAI1*, *PTGER4*, *FBXO34*; fig. 6 and supplementary fig. S4, Supplementary Material online), though the other codon positions appear unsaturated and are still accumulating raw pairwise distance. Even the third codon position of some of the most rapidly evolving genes (e.g., *ASXL1*, *BRCA1*, *SETX*) show linearly increasing divergence up to the oldest divergence times at ∼200 Ma. In comparison, the third codon position for mitochondrial *ND2* sequences from each taxon shows rapid substitution saturation, as expected, by ∼60 Ma (see fig. 6). Given the limited evidence for saturation even at divergences spanning all squamates, we expect that most researchers will not encounter any substitution saturation in RELEC loci at the moderate evolutionary scales usually encountered in the majority of phylogenomic studies. The phylogenetic informativeness profiles suggest that RELEC loci peak in informativeness between ∼25 and 150 Ma (fig. 4), and we therefore recommend these scales as the regions where RELEC loci would be most effective. Still, we do not expect slight third codon position saturation to strongly skew phylogenetic analyses if there is sufficient signal at the other sites.

Exons that have roughly equal rates of evolution at all three codons are unusual (fig. 6*d*) but we observed this pattern for 22 RELEC exons (10%). Typically, third codon positions evolve much faster and this pattern is often observed across large multilocus data sets (e.g., Baker et al. 2014 for 594 protein-coding loci). These loci may be undergoing relaxation of purifying selection and are a reflection of the increased phylogenetic utility of RELEC loci. However, for the entire RELEC data set, the difference in slopes between third position and first and second positions is not significantly correlated with the RF distance from the species tree as might be expected if equal rates at all positions were associated with more accurate gene trees.

### Recombination and Linkage

The chance of a gene carrying a past recombination event increases for longer genes and if recombination occurs can lead to neighboring segments of DNA with different genealogical histories that can skew phylogenetic estimates (Posada and Crandall 2002; Degnan and Rosenberg 2009). A benefit of using UCEs or RADseq is that one may gather large numbers of loci that can be analyzed independently with one SNP per locus, disregarding potential linkage disequilibrium (Leaché and Oaks 2017), but this method would not be appropriate if only capturing a small number of RELEC loci. Transcriptome data with loci that span multiple distant exons are the most likely kind of data to be susceptible to intragenic recombination (Springer and Gatesy 2016) that do indeed violate assumptions of the multispecies coalescent (Edwards et al. 2016). RELEC loci span from ∼1,500 to 12,000 bp in aligned length (table 1), so it is possible that recombination could occur within them, albeit with lower frequency than with transcriptome data that can span hundreds of thousands of bases. Still, we do not expect recombination to be a severe problem for RELEC as recombination events leading to incomplete lineage sorting appear to be rare in real-world data sets (Edwards et al. 2016), and unrecognized intralocus recombination events have been shown to have little effect on summary-coalescent species tree analyses (Lanier and Knowles 2012). We hypothesize that except in rare cases it is more valuable to capture longer genes that contain greater phylogenetic signal (Edwards et al. 2016) and are more likely to recover the true species tree (supplementary fig. S5, Supplementary Material online; Mirarab, Bayzid, et al. 2014; Roch and Warnow 2015).

**Fig. 7.**
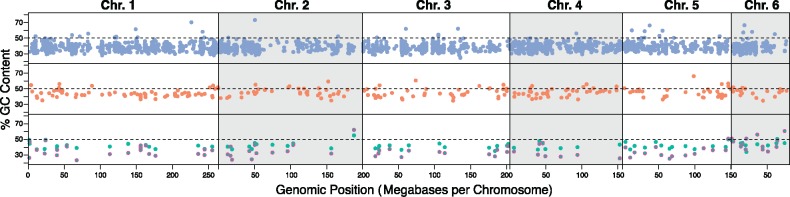
The genomic positions of UCE (top), AHE (middle), and RELEC (bottom) loci on the six *Anolis carolinensis* macrochromosomes. Markers that correspond to microchromosomes or unmapped regions are not shown. The *y* axis shows the % GC content at each locus in *A. carolinensis*, with dots outlined in magenta in the RELEC panel corresponding to third codon GC content. A pattern of GC content increase at chromosome ends (where recombination is highest) would be evidence for GC-biased gene conversion, though this pattern is not apparent in any data set.

We assessed potential linkage of markers by comparing map distances of loci on the *Anolis* macrochromosomes (fig. 7). For RELEC, only in the few cases where two exons were from a single gene (*APOB*, *CSPG4*, *MACF1*, *PHF3*, *TRANK1*) were loci within 50,000 bp of another RELEC locus. In more complex data sets, linkage should perhaps be assessed for these closely spaced loci, but in general, we do not expect linkage to pose a problem for RELEC. In contrast, ∼20% of UCE loci are within 10,000 bp of another UCE locus, and more than half are within 50,000 bp of another. This is likely simply due to the sheer number of UCE probes, but this potential linkage could pose a bias if not accounted for as certain gene tree genealogies from loci in areas of tight linkage may be more common than others and affect species tree analyses. AHE loci show similar map distances as the RELEC loci, but in examining this, we encountered some previously unidentified aspects of the AHE loci. The updated AHE vertebrate probes from version 1 vertebrate (Lemmon et al. 2012) to version 2 removed originally separate markers with overlapping flanking sequencing that would lead to duplicated data and also attempted to combine multiple AHE loci on a single gene into one locus (Prum et al. 2015; Ruane et al. 2015; Tucker et al. 2016), however, there remain several cases of multiple AHE loci on a single gene (supplementary table S2, Supplementary Material online). For example, in version 2, we found 11 independent AHE loci representing multiple regions of a single gene, *TTN*, spanning over 240,000 bp in length. Furthermore, *FAT4* and *RHOV* each have three AHE markers representing these genes, and 12 other genes have two AHE markers associated with them. In total, 41 of the AHE version 2 vertebrate loci show duplicates, and many of the targets are within 10,000 bp of each other on the *Anolis* chromosomes (see supplementary table S2, Supplementary Material online). It is therefore important that studies utilizing AHE do not treat closely separated loci independently and are careful to account for potential linkage or functional similarity. We also found using BLAST searches to the www.ensembl.org database (last accessed November 24, 2019) that the proportion of target regions containing coding sequence has increased from 90% in version 1 (Lemmon et al. 2012) to 98% in version 2 (this study; probes from Ruane et al. 2015), suggesting that it is now primarily a protein coding data set yet studies have not utilized known rate differences among codon positions in evolutionary models or partitioning schemes.

### Coding versus Noncoding Markers

There has been speculation that phylogenomic analysis of exons may be misleading due to biased gene conversion causing genomic-scale molecular convergence of protein-coding sequences (Jarvis et al. 2014). Jarvis et al. (2014) proposed that potentially misleading phylogenetic results in birds may be due to GC-biased gene conversion that acts most strongly in highly recombining regions, as they found rapidly evolving genes near the ends of chromosomes had increased GC content. However, Reddy et al. (2017) rejected this hypothesis by showing that the discordance between coding and noncoding data sets was likely caused by data-type effects due to violation of models, and GC-bias has been shown to lead to gene tree incongruence in noncoding data sets as well (Bossert et al. 2017). Still, the problem of GC-biased gene conversion is less likely to occur in squamates because squamates show decreased GC3-content heterogeneity compared with other amniotes (Fujita et al. 2011; Figuet et al. 2014). We plotted GC and GC3 content variation for loci between the three data sets on the *A.* *carolinensis* macrochromosomes (fig. 7). While there does exist some variation in GC content, it is scattered across the chromosomes, and we do not observe a pattern of increased GC content (or third codon GC content) at the chromosome ends where recombination rates are highest and GC-biased gene conversion tends to occur. As biased gene conversion has been implicated in many systems and can negatively impact phylogenetic reconstruction (Marais 2003; Gruber et al. 2007), future studies should implement alternate models that allow for base frequency nonstationarity and should assess model fit in both coding and noncoding regions (Lockhart et al. 1994; Gowri-Shankar and Rattray 2007).

Despite concerns that selection on protein-coding regions could lead to misleading phylogenetic relationships, we emphasize that selection is not necessarily more intense on coding sequences relative to other genomic regions. For example, introns likely undergo relaxed selection (Chamary et al. 2006) while UCEs must be undergoing strong purifying selection to remain conserved across such distant groups (Katzman et al. 2007). Gene-averaged d*N*/d*S* ratios provide a rough measure of selection intensity on coding regions (though site-specific ratios are more appropriate for searching for more detailed evidence of selection). We found that RELEC loci in general undergo purifying selection to near neutral evolution, with per-locus mean d*N*/d*S* ratios usually <1 (mean = 0.48; sd = 0.19; range = 0.16–1.08). A higher nonsynonymous rate for RELEC markers as opposed to average rates across coding sequences in other organisms (0.15–0.3; Jordan et al. 2004; Buschiazzo et al. 2012) suggests relaxed purifying selection is common in RELEC markers, which likely partially accounts for their faster evolutionary rates. This ability to model nucleotide evolution and the strength of selection (McDonald and Kreitman 1991) is a distinct advantage of using protein-coding DNA sequences for phylogenetics. Rate differences among first, second, and third codon positions, for example, are a well-understood biproduct of selection acting more strongly against nonsynonymous versus synonymous mutations (Jackman et al. 1999) and this among-site rate variation in coding sequences may be beneficial to phylogenetic reconstruction (Klopfstein et al. 2017). Translations to amino acid sequences are also commonly used for phylogenetic analysis (supplementary fig. S3, Supplementary Material online) and detailed codon models can accurately parameterize for rate differences between nonsynonymous and synonymous substitutions or between all codons (Yang 2007), and can now be rapidly implemented in recent versions of IQtree (Nguyen et al. 2015). Finally, relaxed-clock models can allow for accurate phylogenetic reconstruction by accommodating for lineage-specific evolutionary rate differences. Analyses of RELEC data sets will benefit by making use of our increased understanding of protein evolution and may allow for greater confidence in phylogenetic hypotheses.

### Biological Processes

**Fig. 8.**
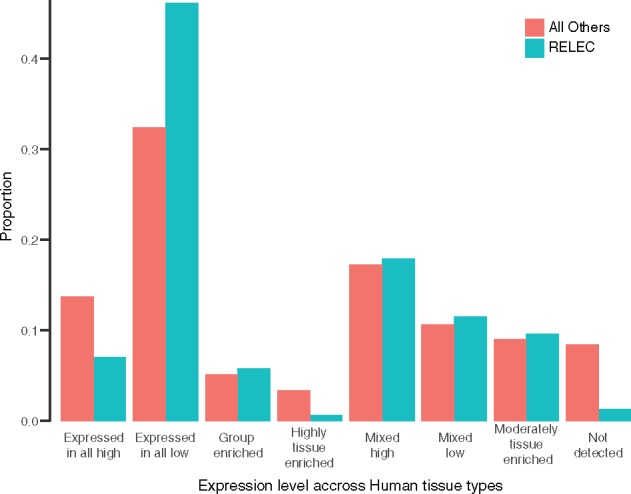
Comparison of expression level categories of RELEC genes to background levels in human tissues. RELEC genes tend to be expressed in all tissues at low rates, while fewer are expressed in all tissues at high rates (χ^2^=27.3; *P* = 0.00029).

There is unlikely to be a unifying functional role of RELEC loci, nor one single process that maintains long exons that also rapidly change. We searched for functional patterns among RELEC genes by investigating expression levels across human tissues (data of Fagerberg et al. 2014). We found that RELEC genes are expressed in humans at significantly different levels compared with the background of all genes (see fig. 8; χ^2^ = 27.3; *P* = 0.00029). Specifically, the largest proportional category of RELEC genes (46.2%) are those that are expressed in low levels across all tissues, over a background level of (32.4%), while very few RELEC genes are expressed at high levels across all tissues (7.0%), compared with a higher background rate (13.8%). Furthermore, almost none (0.6%) of the RELEC genes are highly tissue enriched. These results are interesting as widely expressed genes tend to have slower substitution rates when compared with tissue-specific genes (Zhang and Li 2004), so one may expect the rapidly evolving RELEC genes to be tissue-specific. However, it is important to note that RELEC genes are not the fastest evolving genes in the genome, but the fastest that still maintain very long exons. These patterns suggest that in general RELEC genes function as important proteins that are used in all tissue types, with low expression rates loosening constraints on substitution rate (Pál et al. 2001), but broad expression across all tissue types may lead to maintenance of the size of the longest exon and the corresponding protein. An important caveat of this analysis is that some RELEC gene isoforms (in humans) are much shorter and do not contain the long RELEC exon we selected for phylogenomics. Therefore, the isoform containing the RELEC exon may not be expressed as broadly as shorter isoforms of the gene, and the long isoform may serve a more specific function. Other exceptions to these patterns are genes that are only expressed briefly in development and/or in limited tissue types. For example, *ENAM* (enamelin), encodes the largest protein of the enamel matrix and is only expressed during tooth growth (https://ghr.nlm.nih.gov/gene/ENAM; last accessed November 24, 2019). *RAG1* is involved in immune response and is only expressed in lymphocytes, and likely evolves rapidly to facilitate binding to changing substrates (https://ghr.nlm.nih.gov/gene/RAG1; last accessed November 24, 2019).

We also assessed enrichment of Gene Ontology (GO) functional categories of the RELEC genes as a whole on the PANTHER classification system. We found significant overrepresentation of RELEC genes for three molecular function categories associated with binding: tubulin binding (13 hits), DNA binding (41 hits), and ion binding (77 hits), which together constituted for more than half (91) of the RELEC genes after accounting for duplicates. The enrichment for binding suggests that the long RELEC proteins might be subject to rapid change in amino acid sequence in order to complete their designated binding task. For example, the *BRCA1* and *BRCA2* genes are well-studied and known to be used to repair damaged DNA (Moynahan et al. 1999, 2001), and we speculate that this function may also require rapid amino acid replacement to account for different kinds of DNA damage. We also found significant overrepresentation in several biological process categories, including microtubule cytoskeleton organization (19 hits), organelle localization (18 hits), cell cycle process (24 hits), and developmental process (73 hits), accounting for 92 unique genes. Microtubule organization, in particular, is interesting as it often requires a protein to bind to both the microtubule and another location such as the cytoskeleton, plasma membrane, or other molecules (e.g., *MACF1*, *MAP1A*, *MAP1B*, *MAP2*). We assessed the number of protein interactors for each RELEC gene on BioGRID, hypothesizing that RELEC genes might have fewer protein interactors because more interactions might act as a purifying selective pressure. We found that numbers of protein interactors ranged from zero or one to several hundred, with no distinct pattern emerging (supplementary table S1, Supplementary Material online), suggesting that there are many possible functional roles that can still permit rapid evolution. It is important that sets of phylogenomic loci do not all contribute to the same function or pathway, as this might lead to linked evolutionary histories among loci that could bias phylogenetic estimates. Our functional analyses clearly show that RELEC encoded proteins provide a wide variety of biological functions, and therefore are unlikely to be susceptible to this bias.

### Practical Implementation

Applying RELEC to any amniote group is relatively straightforward. Probes can be designed from transcriptomes or genomes and can be sequenced using many available methods (e.g., Illimuna and Fluidigm). Target enrichment for conserved long exons among divergent taxa has been well-established in numerous studies (Bi et al. 2012; Li et al. 2013). We are currently in the process of using Illumina sequencing for RELEC loci in diplodactylid geckos using probes designed from *Correlophus ciliatus*. We also extracted the target sequences from the *Gallus gallus* genome for use in avian phylogenomics. For the geckos, we developed custom biotinylated RNA bait libraries using the MYBaits target enrichment system (MYcroarray, Ann Arbor, Michigan). Based on the 179 RELEC exons, we designed ∼10,000 probes consisting of 120 bp baits with 60 bp overlap between baits, targeting exons 120 bp or larger. The baits were filtered for repetitive sequences by MYcroarray using RepeatMasker (http://www.repeatmasker.org/; last accessed November 24, 2019) and compared with the entire *Correlophus* genome to ensure unique binding of baits. Our procedure for capturing RELEC baits follows the detailed protocol for exon capture in sharks (Li et al. 2013) which captures divergent sequences by both decreasing the temperature of hybridization successively and increasing the time of hybridization of the baits to the target. The use of RELEC loci in future studies would involve designing a set of RELEC bait libraries based on the genome of a member or close relative of the group of interest. We extracted the RELEC loci from several mammal and bird genomes, and provide these sequences for users to build probes or to use them to extract RELEC loci from other genomes using custom scripts (https://github.com/benrkarin/RELEC).

It is possible that substantial genetic divergences arising from rapidly evolving markers could lead to poor hybridization efficiency in distant taxa. Though drop-off in exon capture efficiency for divergent taxa has been shown to occur in some experiments (Bi et al. 2012; Bragg et al. 2016), it is common for sequences to still be effectively captured at high enough levels for phylogenetic analysis even across extremely divergent taxa (Li et al. 2013; Ilves and López-Fernández 2014; Portik et al. 2016; Bragg et al. 2018). By coupling tiling across these relatively long probe regions and sequencing longer read lengths (e.g., MiSeq paired-end 300 bp reads), we expect to maintain capture efficiency for the RELEC markers even at substantial genetic divergences.

As it is becoming easier and less expensive to sequence full genomes or very large sets of loci, we expect that RELEC loci will most commonly be applied to new systems in two ways: 1) targeted directly using bait capture, possibly in conjunction with other sets of loci, or 2) extracted from whole genomes as a unique marker set for comparison with other sets. With the bait capture approach, we expect that RELEC loci will often be targeted in combination with other exons of known functional interest, and often in combination with other sets such as the Squamate Conserved Loci (SqCL) probes of Singhal et al. (2017), which combine AHE, UCEs, and other commonly used squamate markers (Wiens et al. 2012), and CNEEs (Edwards et al. 2017). If a strongly supported phylogeny is the primary goal of a study, these reduced representation data sets are the most cost-effective strategy. If the researcher has other questions, such as phenotype–genotype associations, and genome architecture, then whole genomes can provide the means to investigate these topics and also extract different loci sets, such as RELEC, for estimating the phylogeny of the group. When whole genomes are sequenced, RELEC loci may be used as a preferred set that can be run as a whole using intensive full Bayesian coalescent species tree methods that are powerful at dealing with incomplete lineage sorting, as we have done in this study.

### Conclusion

The recent acceleration in the generation of full genomes has allowed for the development of unique sets of markers that can be tailored for particular research questions in nonmodel organisms. RELEC is one such example of a set of loci primarily intended to be capable of resolving difficult nodes in the tree of life, focusing on maximizing phylogenetic signal while maintaining orthology across evolutionary scales and remaining computationally tractable for comprehensive phylogenetic analyses. In the future, choosing an appropriate strategy for selecting sets of sequences to make reliable gene trees will become increasingly important. As more whole genomes are sequenced and published, our approach to finding and utilizing long stretches of comparable coding sequences can be used effectively to generate a set of reliable gene trees. We look forward to future phylogenomic studies that will assess the utility of RELEC loci at resolving difficult nodes.

## Materials and Methods

We generated and tested the set of RELEC markers for an amniote data set under the following criteria: 1) Exons at least 1,500 bp in length (with one exception, *OMG*); 2) Rapidly evolving with the raw percent divergence greater than or equal to that of *RAG1* for both deep and shallow splits (calculated by comparing *A.* *carolinensis* with *Gallus gallus* and *Python molurus*). *RAG1* has shown utility for reconstructing phylogenies at both deep and shallow levels (Groth and Barrowclough 1999) and has become one of the most commonly used nuclear markers in vertebrates (Barker et al. 2004; Townsend et al. 2004; Gruber et al. 2007; Hugall et al. 2007; Fuchs et al. 2011; Portik et al. 2011; Pyron 2011; Gamble et al. 2012; Harrington and Near 2012; Gartner et al. 2013); 3) Presence in all amniote groups (confirmed in mammals, birds, and squamates with a few exceptions); and 4) Single copy, with paralogs only allowed for duplications that predate the common ancestor minimally of squamates (though in most markers it predates the common ancestor of all amniotes).

We identified many of the RELEC loci by querying the Ensembl database (Aken et al. 2017) for exons in *A.* *carolinensis* and other genomes. However, annotation errors are common on Ensembl especially for long exons (such as premature or incorrect boundaries or missing annotations) so we individually considered each locus to assess correct annotation and other criteria. We gathered additional exons by querying and inspecting all long mammalian exons on the Orthomam database (Douzery et al. 2014) and cross comparing the results on Ensembl for *Anolis* and across the amniote tree using BLAST searches. For example, for the 43 RELEC exons that are 4,000 bp and longer, 27 were annotated by Ensembl on the *Anolis* genome and were easily identified, and 16 more were identified following searches on Orthomam and were not originally found because they were missing annotations or had errors on Ensembl. Using these methods, we are confident that we have gathered all or nearly all single copy exons that show this length and evolutionary rate and are also present among amniotes.

To avoid issues of gene duplications, we limited the set of loci in the RELEC data set to those that were present in all amniote groups, and only included genes with known paralogs if duplications occurred on very deep lineages (e.g., for all mammals) and had been maintained. We also excluded some exons that present high levels of repeats that could bias phylogenetic analyses (e.g., *TTN*). Unlike other rapidly evolving regions of the genome that may mutate beyond recognition over deep time scales, exons are much more readily maintained across distantly related organisms, likely due to their direct functional role. For example, the major exons for the rapidly evolving genes *BRCA1* and *EXPH5* can be aligned and compared across all amniotes, whereas the intervening introns have accumulated substantial indels (see www.ensembl.org gene tree browser). After assembling the set of loci, we checked for presence and proper annotation of RELEC loci across six major tetrapod lineages using the chicken, saltwater crocodile, painted turtle, *Anolis*, human, and *Xenopus* genomes. We did this first by querying Ensembl gene names to extract annotation information for the longest exon. However, many genes did not have proper names on Ensembl, and for these, we manually used TBlastN searches on Ensembl to confirm presence. In these cases, we noted poor annotations when the exon or gene was either not annotated at all or had incorrect intron–exon boundaries, and determined the length of unannotated exons by searching for open reading frames surrounding the highest TBlastN hit (supplementary table S4, Supplementary Material online). The final RELEC data set includes 179 exons, representing 173 unique genes (6 genes with two exons each).

### Squamate Genomes

We queried 15 published and 2 unpublished squamate genomes to build the RELEC, UCE, and AHE data sets for comparison in this study. Assembled genomes were downloaded from NCBI(_*1*_), GigaScience(_*2*_), or unpublished(_*3*_). This includes the snakes *Python* *molurus_1_* (Castoe et al. 2013), *Boa constrictor_2_* (Bradnam et al. 2013), *Vipera berus_1_*, *Crotalus horridus_1_*, *Crotalus* *mitchellii_1_* (Gilbert et al. 2014), *Deinagkistrodon acutus_2_* (Yin et al. 2016), *Protobothrops mucrosquamatus_1_* (Aird et al. 2017), *Ophiophagus hannah_1_* (Vonk et al. 2013), *Thamnophis sirtalis_1_*(Perry et al. 2018), and *Pantherophis guttatus_1_* (Ullate-Agote et al. 2014), and the lizards *Gekko japonicus_1_*(Liu et al. 2015), *E.* *macularius_2_* (Xiong et al. 2016), *C.* *ciliatus_3_*, *Christinus marmoratus_3_*, *D.* *gracilis_2_* (Song et al. 2015), *P.* *vitticeps_2_* (Georges et al. 2015), and *A.* *carolinensis_1_* (Alföldi et al. 2011). At the time of data acquisition, three more squamate species genomes were available for *Thamnophis elegans* (Vicoso et al. 2013)*, Sistrurus miliarus* (Vicoso et al. 2013), and *Sceloporus occidentalis* (Genomic Resources Development Consortium et al. 2015), though due to short read lengths and/or missing data, we chose not to use them (the purpose of these studies was not to acquire the high coverage or long read lengths needed for our purposes). The genomes of other squamates were published after our analyses were completed (*Shinisaurus crocodilurus*, Gao et al. 2017; *Salvator merianae*, Roscito et al. 2018; *Lacerta viridis* and *Lacerta* *bilineata*, Kolora et al. 2019; *Varanus komodoensis*, Lind et al. 2019; *Zootoca vivipara*, Yurchenko et al. 2019) and we were not able to include them. The genomes we used encompass several divergent squamate lineages, but are primarily focused on snakes and geckos. Since geckos represent the sister clade to remaining squamates (Streicher and Wiens 2017), the data set provides satisfactory depth for comparisons of the different methods.

### Data Generation and Alignments

We extracted and aligned sequences from the 17 genomes using customized scripts and using similar (though slightly adjusted) methods for each data set. To extract both the RELEC and AHE loci from the genomes, we employed the BLAST+ command line tools (Camacho et al. 2009) to build local BLAST databases for each genome assembly. We developed the initial data set for the RELEC loci with the genome of the gecko *C.* *ciliatus*. After confirming the translation frame visually, we translated the sequences using the program Geneious v9.0.4 (Kearse et al. 2012), and then used the *TBlastN* command from the BLAST+ toolkit to search for similar amino acid sequences in any frame in each genome. This method allowed for proper identification and extraction of orthologous exon regions even for rapidly evolving genes and the most divergent lineages. For the AHE data set, we searched the genomes using the 389 version 2 vertebrate loci from Ruane et al. (2015). Since AHE markers include a combination of coding and noncoding regions, and because there is no detailed list of which markers are associated with specific genomic regions, we used the nontranslated *BlastN* search algorithm for each AHE search sequence.

BLAST searches for highly divergent sequences often produce multiple segmented hits for a single locus, rather than the entire length of the marker. In order to extract the entire orthologous sequence for each locus, we designed a script, Ortholog Assembly and Concatenation (OAC), in R v3.1.13 (R Core Team 2016) to combine coordinates of sequences that had more than one BLAST hit within close proximity on a single assembled contig, and extract and align the new sequence with a flanking region that was later trimmed to the initial query sequence (available at https://github.com/benrkarin/RELEC). This ensured that the maximum hit sequence length would be captured despite potential indels causing segmentation of BLAST hits to the same contig. Though on occasion some sequences were clearly split into multiple assembled contigs, we refrained from extracting the whole sequence to eliminate the possibility of unintentionally capturing paralogous sequences from different genomic regions. We indexed the genome assemblies using SAMtools (Li et al. 2009), and extracted the sequences based on the combined BLAST search coordinates for each marker with the *faidx* command. We then aligned individually extracted sequences by locus using MAFFT v7.130b (Katoh and Standley 2013), allowing the program to automatically determine sequence direction to accommodate for reversed sequences. All sequence alignments for RELEC and AHE were visually inspected in Geneious to confirm successful sequence capture and manually trimmed to reduce ragged ends. We conducted additional BLAST searches for any missing sequences, and to confirm, we had captured the maximal sequence length available in the genomes. We conducted further BLAST searches of RELEC loci extracted from the *C.* *ciliatus* genome against a combined transcriptome for *C. ciliatus* generated from six tissue types (eye, brain, tail, testis, ovary, whole embryo). We found transcripts corresponding to all RELEC loci in the transcriptome, confirming that RELEC genes are actually transcribed into mRNA.

For RELEC, we allowed incomplete alignments with less than the total 17 taxa for four exons: *PKDREJ_B*, which is absent in gekkotans and *D.* *gracilis*, but is a very rapidly evolving and informative marker that will be useful in shallow-scale phylogenetic studies; *TRANK1* exons 1 and 2, which are absent in the genomes of *O.* *hannah*, *T.* *sirtalis*, and *Pantherophis* *guttatus*, and partially deleted in *V.* *berus*; and *NAIP*, which is absent from the *P.* *vitticeps* genome. For AHE, we only used completely sampled 17-taxa alignments.

For the UCE data set, we followed the *phyluce* software package manual (Faircloth 2016) using the provided *Anolis* 5k probeset (available from https://github.com/faircloth-lab/phyluce; last accessed November 24, 2019), and extracted a flanking region of 300 bp for each UCE marker. This flanking region is comparable to mean aligned sequence lengths generated in empirical studies (e.g., Crawford et al. 2015: 820 bp; Grismer et al. 2016: 645 bp). For the UCE data set, we used a complete matrix containing data for all 17 taxa for the main analysis (1,517 loci), but also generated data sets for loci with at least 15 taxa (additional 1,151 loci) and at least 9 taxa (additional 741 loci) for comparison (see supplementary fig. S8, Supplementary Material online, other results are comparable to the complete set and are not shown).

We used reciprocal BLAST searches to assess overlap between data sets. We found that 17 AHE loci match UCE loci; none of the RELEC loci matches any UCE loci; and 28 RELEC loci match AHE loci (4 of which are from Wiens et al. 2012). Though for these overlapping regions it is important to note that RELEC targets the entire exon rather than a portion of it or the flanking intron.

### Phylogenetic Analyses

We conducted summary-coalescent species tree analyses using ASTRAL-III (Zhang et al. 2018) based on sets of 100 maximum likelihood bootstrap replicates generated on each locus individually in RAxML v8.1.15 (Stamatakis 2014), and specified all four of the gekkotans as the rooting taxa. We chose the GTRGAMMA model for all analyses, as Stamatakis (2015) suggests that other models may be inappropriate for data sets with relatively few taxa such as this one. We also generated gene trees for RELEC loci translated to amino acid sequences in RAxML, allowing the program to choose the model with the PROTGAMMAAUTO setting. We conducted a final ASTRAL analysis on a combined data set of all AHE, UCE, and RELEC loci (2,016 loci after excluding any overlapping loci). We also conducted parallel analyses in IQTree (Nguyen et al. 2015) allowing for automatic model selection (Kalyaanamoorthy et al. 2017) and recovered nearly identical results for gene tree and ASTRAL estimates (not shown).

Concatenated data sets and partition files were generated using the perl program, BeforePhylo v0.9.0 (Zhu 2014), and concatenated maximum likelihood trees were generated with 1,000 bootstrap replicates with RAxML, under the same rooting and model settings as the gene trees. To maintain consistency across data sets, we partitioned the concatenated analyses by each locus, without specifying different partitions for codon positions in the RELEC data set. To improve computing time, we ran the concatenated analyses on the CIPRES Science Gateway (Miller et al. 2010).

We analyzed concordance and accuracy of the maximum likelihood gene trees in R by incorporating functions from the *ape* (Paradis et al. 2004) and *phangorn* (Schliep 2011) packages to compute the proportion of the highest scoring maximum likelihood trees in each data set that display a particular node in the species tree. This is similar to the gene concordance factor that can be estimated in IQTree (Minh et al. 2018) (results in supplementary table S3, Supplementary Material online), but our technique uses a different rooting method that allows for a proportional value to be calculated at every node, whereas the concordance factor excludes some nodes. We also examined the Robinson–Foulds (RF) distance between the best scoring individual gene trees and the species tree. RF distances quantify the accuracy of gene trees, with values of 0 corresponding to an exact match, a value of 2 corresponding to one node difference, and so on. To examine the accuracy of gene trees at a finer scale, we conducted the same analysis restricted to the eight colubroid snakes in the tree. We assessed the robustness of gene trees to correctly build the species tree (using the data set-specific ASTRAL species tree to reduce bias) by iteratively running ASTRAL on randomly selected jackknife replicate bins of varying numbers of gene trees from two gene trees to the full number in each data set using a python script provided by Daniel Portik.

We used the multispecies coalescent to estimate species trees from the complete set of 179 RELEC loci using StarBEAST2 (Ogilvie et al. 2017), part of the BEAST2 package (Bouckaert et al. 2014). This is a new implementation of the *BEAST method (Heled and Drummond 2010) but is considerably faster and enables the use of dozens or hundreds of loci. We used the analytical population size integration, with a strict molecular clocks and general time reversible (GTR) plus gamma model for each locus. We initiated two analyses for 50 million generations each with a pre burn-in of 1 million generations, sampling every 5,000 generations. The program Tracer v1.7.1 (Rambaut et al. 2018) was used to visually assess stationarity and convergence between runs and determine the burn-in. The posterior distribution of post burn-in species trees was visualized using DensiTree v2.2.2 (Bouckaert and Heled 2014). BEAST analyses were conducted on a high performance computer with 32 2.4 GHz processing cores and 512 GB RAM running CentOS 7.

### Summary Statistics

We computed general summary statistics (see supplementary table S1, Supplementary Material online) for each marker in R using the packages *ape* (Paradis et al. 2004), *phyloch* (Heibl 2016), and *adephylo* (Jombart and Dray 2016). These included the GC content across all sites, GC content at codon position 3 (GC3), average pairwise identity for alignments, proportion of variable and parsimony informative sites, and raw pairwise genetic distance between *Gekko japonicus* and *A.* *carolinensis* as a rough relative measure of evolutionary rate (supplementary table S1, Supplementary Material online). We also calculated the number of segregating sites (supplementary fig. S5, Supplementary Material online), and assessed the size and width of alignment gaps (supplementary fig. S6, Supplementary Material online).

### Phylogenetic Informativeness and Substitution Saturation

We profiled the phylogenetic informativeness (Townsend 2007) of each marker using the web-based tool PhyDesign (López-Giráldez and Townsend 2011), specifying the DNArates algorithm. This method provides an estimate of the relative power of each locus to resolve a given node in the tree. The program requires a timetree as input, so we transformed the output ASTRAL phylogeny (AHE, UCE, and RELEC combined data set topology) into a timetree using the *ape* package and *ScalePhylo* (Hunt 2011) script in R. *ScalePhylo* allowed us to force a time-calibration on the nodes of the tree based on input divergence dates, which we acquired from the date estimate for each pair of taxa from http://timetree.org/ (last accessed November 24, 2019) (Hedges et al. 2015). We downloaded the output from the PhyDesign web portal, and plotted the measures of phylogenetic informativeness for each data set separately in R to allow for comparison between data sets. We visually examined substitution saturation in the RELEC loci by generating plots of raw sequence divergence at divergence date estimates from the http://timetree.org/ database. We used the output in R to compare how maximum informativeness scales with alignment length, and also the time of maximum informativeness. We also scaled the curves by alignment length in order to assess the informativeness independent of length (results shown in supplementary fig. S7, Supplementary Material online).

### Biological Processes

We sought to investigate if RELEC loci are subject to unique biological processes that contribute to their rapid evolution and maintenance of long exon length. We compared the expression levels across human tissues using the transcript abundance categorization of Fagerberg et al. (2014), and also compared expression of RELEC genes relative to the background within individual tissue types. We searched the Swiss-Prot database (http://www.uniprot.org; last accessed November 24, 2019) for the number of GO terms associated with each protein for *Homo sapiens* from any of the three GO categories (biological process, molecular function, and cellular component). This gives an approximation of the breadth of functional utility for each protein, and may potentially influence the rate of molecular evolution. We also searched the BioGRID database (http://thebiogrid.org; last accessed November 24, 2019) for the number of unique protein interactors associated with each protein in *Homo sapiens*, for which the data are most extensive. Finally, we looked for GO term statistical overrepresentation for human genes using the web server for the PANTHER 14.1 classification system (http://pantherdb.org; last accessed November 24, 2019), using Fisher’s exact test and a Bonferroni correction for multiple testing. 

## Data Availability

Scripts, sequence alignments, and other resources for mammals, birds, and squamates are available at https://github.com/benrkarin/RELEC.

## Supplementary Material

msz263_Supplementary_Data

## References

[msz263-B1] Abascal F, Zardoya R, Telford MJ. 2010 TranslatorX: multiple alignment of nucleotide sequences guided by amino acid translations. Nucleic Acids Res. 38(Suppl 2):W7–W13. 20435676 10.1093/nar/gkq291 PMC2896173

[msz263-B2] Aird SD, Arora J, Barua A, Qiu L, Terada K, Mikheyev AS. 2017 Population genomic analysis of a pitviper reveals microevolutionary forces underlying venom chemistry. Genome Biol Evol. 9(10):2640–2649. 29048530 10.1093/gbe/evx199 PMC5737360

[msz263-B3] Aken BL, Achuthan P, Akanni W, Amode MR, Bernsdorff F, Bhai J, Billis K, Carvalho-Silva D, Cummins C, Clapham P. 2017 Ensembl 2017. Nucleic Acids Res. 45(D1):D635–D642. 27899575 10.1093/nar/gkw1104 PMC5210575

[msz263-B4] Alföldi J, Di Palma F, Grabherr M, Williams C, Kong L, Mauceli E, Russell P, Lowe CB, Glor RE, Jaffe JD. 2011 The genome of the green anole lizard and a comparative analysis with birds and mammals. Nature 477(7366):587–591. 21881562 10.1038/nature10390 PMC3184186

[msz263-B5] Arcila D, Ortí G, Vari R, Armbruster JW, Stiassny MLJ, Ko KD, Sabaj MH, Lundberg J, Revell LJ, Betancur-R R. 2017 Genome-wide interrogation advances resolution of recalcitrant groups in the tree of life. Nat Ecol Evol. 1(2):1–10. 28812610 10.1038/s41559-016-0020

[msz263-B6] Baker AJ, Haddrath O, Mcpherson JD, Cloutier A. 2014 Genomic support for a moa-tinamou clade and adaptive morphological convergence in flightless ratites. Mol Biol Evol. 31(7):1686–1696. 24825849 10.1093/molbev/msu153

[msz263-B7] Barker FK, Cibois A, Schikler P, Feinstein J, Cracraft J. 2004 Phylogeny and diversification of the largest avian radiation. Proc Natl Acad Sci U S A. 101(30):11040–11045. 15263073 10.1073/pnas.0401892101 PMC503738

[msz263-B8] Bejerano G, Pheasant M, Makunin I, Stephen S, Kent WJ, Mattick JS, Haussler D. 2004 Ultraconserved elements in the human genome. Science 304(5675):1321–1325. 15131266 10.1126/science.1098119

[msz263-B9] Betancur-R R, Naylor GJP, Ortí G. 2014 Conserved genes, sampling error, and phylogenomic inference. Syst Biol. 63:257–262. 24293251 10.1093/sysbio/syt073

[msz263-B10] Bi K, Vanderpool D, Singhal S, Linderoth T, Moritz C, Good JM. 2012 Transcriptome-based exon capture enables highly cost-effective comparative genomic data collection at moderate evolutionary scales. BMC Genomics 13(1):403. 22900609 10.1186/1471-2164-13-403 PMC3472323

[msz263-B11] Blom MPK, Bragg JG, Potter S, Moritz C. 2017 Accounting for uncertainty in gene tree estimation: summary-coalescent species tree inference in a challenging radiation of Australian lizards. Syst Biol. 66(3):352–366. 28039387 10.1093/sysbio/syw089

[msz263-B12] Bossert S, Murray EA, Blaimer BB, Danforth BN. 2017 The impact of GC bias on phylogenetic accuracy using targeted enrichment phylogenomic data. Mol Phylogenet Evol. 111:149–157. 28390323 10.1016/j.ympev.2017.03.022

[msz263-B13] Bouckaert R, Heled J. 2014 DensiTree 2: seeing trees through the forest. *bioRxiv*: 012401.

[msz263-B14] Bouckaert R, Heled J, Kühnert D, Vaughan T, Wu CH, Xie D, Suchard MA, Rambaut A, Drummond AJ. 2014 BEAST 2: a software platform for Bayesian evolutionary analysis. PLoS Comput Biol. 10(4):e1003537. 24722319 10.1371/journal.pcbi.1003537 PMC3985171

[msz263-B15] Bradnam KR, Fass JN, Alexandrov A, Baranay P, Bechner M, Birol I, Boisvert S, Chapman JA, Chapuis G, Chikhi R, et al 2013 Assemblathon 2: evaluating de novo methods of genome assembly in three vertebrate species. GigaScience 2(1):31. 10.1186/2047-217X-2-10 PMC3844414 23870653

[msz263-B16] Bragg JG, Potter S, Afonso Silva AC, Hoskin CJ, Bai BYH, Moritz C. 2018 Phylogenomics of a rapid radiation: the Australian rainbow skinks. BMC Evol Biol. 18(1):12. 10.1186/s12862-018-1130-4 PMC5800007 29402211

[msz263-B17] Bragg JG, Potter S, Bi K, Moritz C. 2016 Exon capture phylogenomics: efficacy across scales of divergence. Mol Ecol Resour. 16(5):1059–1068. 26215687 10.1111/1755-0998.12449

[msz263-B18] Brandley MC, Bragg JG, Singhal S, Chapple DG, Jennings CK, Lemmon AR, Lemmon EM, Thompson MB, Moritz C. 2015 Evaluating the performance of anchored hybrid enrichment at the tips of the tree of life: a phylogenetic analysis of Australian Eugongylus group scincid lizards. BMC Evol Biol. 15(1):62. 25880916 10.1186/s12862-015-0318-0 PMC4434831

[msz263-B19] Breinholt JW, Kawahara AY. 2013 Phylotranscriptomics: saturated third codon positions radically influence the estimation of trees based on next-gen data. Genome Biol Evol. 5(11):2082–2092. 24148944 10.1093/gbe/evt157 PMC3845638

[msz263-B20] Brown JM, Thomson RC. 2017 Bayes factors unmask highly variable information content, bias, and extreme influence in phylogenomic analyses. Syst Biol. 66(4):517–530. 28003531 10.1093/sysbio/syw101

[msz263-B21] Bryson RW, Faircloth BC, Tsai WLE, McCormack JE, Klicka J. 2016 Target enrichment of thousands of ultraconserved elements sheds new light on early relationships within New World sparrows (Aves: Passerellidae). Auk 133(3):451–458.

[msz263-B22] Buschiazzo E, Ritland C, Bohlmann J, Ritland K. 2012 Slow but not low: genomic comparisons reveal slower evolutionary rate and higher d*N*/d*S* in conifers compared to angiosperms. BMC Evol Biol. 12(1):8. 22264329 10.1186/1471-2148-12-8 PMC3328258

[msz263-B23] Camacho C, Coulouris G, Avagyan V, Ma N, Papadopoulos J, Bealer K, Madden TL. 2009 BLAST+: architecture and applications. BMC Bioinformatics 10(1):421. 20003500 10.1186/1471-2105-10-421 PMC2803857

[msz263-B24] Castoe TA, de Koning APJ, Hall KT, Card DC, Schield DR, Fujita MK, Ruggiero RP, Degner JF, Daza JM, Gu W, et al 2013 The Burmese python genome reveals the molecular basis for extreme adaptation in snakes. Proc Natl Acad Sci U S A. 110(51):20645–20650. 24297902 10.1073/pnas.1314475110 PMC3870669

[msz263-B25] Castoe TA, de Koning APJ, Kim H-M, Gu W, Noonan BP, Naylor G, Jiang ZJ, Parkinson CL, Pollock DD. 2009 Evidence for an ancient adaptive episode of convergent molecular evolution. Proc Natl Acad Sci U S A. 106(22):8986–8991. 19416880 10.1073/pnas.0900233106 PMC2690048

[msz263-B26] Chamary JV, Parmley JL, Hurst LD. 2006 Hearing silence: non-neutral evolution at synonymous sites in mammals. Nat Rev Genet. 7(2):98–108. 16418745 10.1038/nrg1770

[msz263-B27] Chen MY, Liang D, Zhang P. 2015 Selecting question-specific genes to reduce incongruence in phylogenomics: a case study of jawed vertebrate backbone phylogeny. Syst Biol. 64(6):1104–1120. 26276158 10.1093/sysbio/syv059

[msz263-B28] Chen MY, Liang D, Zhang P. 2017 Phylogenomic resolution of the phylogeny of laurasiatherian mammals: exploring phylogenetic signals within coding and noncoding sequences. Genome Biol Evol. 9(8):1998–2012. 28830116 10.1093/gbe/evx147 PMC5737624

[msz263-B29] Chiari Y, Cahais V, Galtier N, Delsuc F. 2012 Phylogenomic analyses support the position of turtles as the sister group of birds and crocodiles (Archosauria). BMC Biol. 10(1):14. 10.1186/1741-7007-10-65 PMC3473239 22839781

[msz263-B30] Chifman J, Kubatko L. 2014 Quartet inference from SNP data under the coalescent model. Bioinformatics 30(23):3317–3324. 25104814 10.1093/bioinformatics/btu530 PMC4296144

[msz263-B31] Crawford NG, Faircloth BC, McCormack JE, Brumfield RT, Winker K, Glenn TC. 2012 More than 1000 ultraconserved elements provide evidence that turtles are the sister group of archosaurs. Biol Lett. 8(5):783–786. 22593086 10.1098/rsbl.2012.0331 PMC3440978

[msz263-B32] Crawford NG, Parham JF, Sellas AB, Faircloth BC, Glenn TC, Papenfuss TJ, Henderson JB, Hansen MH, Simison WB. 2015 A phylogenomic analysis of turtles. Mol Phylogenet Evol. 83:250–257. 25450099 10.1016/j.ympev.2014.10.021

[msz263-B33] Degnan JH, Rosenberg NA. 2009 Gene tree discordance, phylogenetic inference and the multispecies coalescent. Trends Ecol Evol. 24(6):332–340. 19307040 10.1016/j.tree.2009.01.009

[msz263-B34] Dickel DE, Ypsilanti AR, Pla R, Zhu Y, Barozzi I, Mannion BJ, Khin YS, Fukuda-Yuzawa Y, Plajzer-Frick I, Pickle CS, et al 2018 Ultraconserved enhancers are required for normal development. Cell 172(3):491–499. 29358049 10.1016/j.cell.2017.12.017 PMC5786478

[msz263-B35] Dornburg A, Townsend JP, Friedman M, Near TJ. 2014 Phylogenetic informativeness reconciles ray-finned fish molecular divergence times. BMC Evol Biol. 14(1):169. 25103329 10.1186/s12862-014-0169-0 PMC4236503

[msz263-B36] Dornburg A, Townsend JP, Wang Z. 2017 Maximizing power in phylogenetics and phylogenomics: a perspective illuminated by fungal big data. In: Townsend JP, Wang Z, editors. Fungal Phylogenetics and Phylogenomics. Cambridge (MA): Academic Press. p. 1–47. 10.1016/bs.adgen.2017.09.007 29153398

[msz263-B37] Douzery EJP, Scornavacca C, Romiguier J, Belkhir K, Galtier N, Delsuc F, Ranwez V. 2014 OrthoMaM v8: a database of orthologous exons and coding sequences for comparative genomics in mammals. Mol Biol Evol. 31(7):1923–1928. 24723423 10.1093/molbev/msu132

[msz263-B38] Edwards SV, Cloutier A, Baker AJ. 2017 Conserved nonexonic elements: a novel class of marker for phylogenomics. Syst Biol. 66(6):1028–1044. 28637293 10.1093/sysbio/syx058 PMC5790140

[msz263-B39] Edwards SV, Xi Z, Janke A, Faircloth BC, McCormack JE, Glenn TC, Zhong B, Wu S, Lemmon EM, Lemmon AR, et al 2016 Implementing and testing the multispecies coalescent model: a valuable paradigm for phylogenomics. Mol Phylogenet Evol. 94:447–462. 26518740 10.1016/j.ympev.2015.10.027

[msz263-B40] Edwards SV. 2009 Is a new and general theory of molecular systematics emerging? Evolution 63:1–19. 19146594 10.1111/j.1558-5646.2008.00549.x

[msz263-B41] Fagerberg L, Hallström BM, Oksvold P, Kampf C, Djureinovic D, Odeberg J, Habuka M, Tahmasebpoor S, Danielsson A, Edlund K, et al 2014 Analysis of the human tissue-specific expression by genome-wide integration of transcriptomics and antibody-based proteomics. Mol Cell Proteomics. 13(2):397–406. 24309898 10.1074/mcp.M113.035600 PMC3916642

[msz263-B42] Faircloth BC. 2016 PHYLUCE is a software package for the analysis of conserved genomic loci. Bioinformatics 32(5):786–788. 26530724 10.1093/bioinformatics/btv646

[msz263-B43] Faircloth BC, McCormack JE, Crawford NG, Harvey MG, Brumfield RT, Glenn TC. 2012 Ultraconserved elements anchor thousands of genetic markers spanning multiple evolutionary timescales. Syst Biol. 61(5):717–726. 22232343 10.1093/sysbio/sys004

[msz263-B44] Figuet E, Ballenghien M, Romiguier J, Galtier N. 2014 Biased gene conversion and GC-content evolution in the coding sequences of reptiles and vertebrates. Genome Biol Evol. 7:240–250. 25527834 10.1093/gbe/evu277 PMC4316630

[msz263-B45] Fuchs J, Chen S, Johnson JA, Mindell DP. 2011 Pliocene diversification within the South American Forest falcons (Falconidae: *Micrastur*). Mol Phylogenet Evol. 60(3):398–407. 21621621 10.1016/j.ympev.2011.05.008

[msz263-B46] Fujita MK, Edwards SV, Ponting CP. 2011 The *Anolis* lizard genome: an amniote genome without isochores. Genome Biol Evol. 3:974–984. 21795750 10.1093/gbe/evr072 PMC3184785

[msz263-B47] Galtier N, Gouy M. 1998 Inferring pattern and process: maximum-likelihood implementation of a nonhomogeneous model of DNA sequence evolution for phylogenetic analysis. Mol Biol Evol. 15(7):871–879. 9656487 10.1093/oxfordjournals.molbev.a025991

[msz263-B48] Gamble T, Greenbaum E, Jackman TR, Russell AP, Bauer AM. 2012. Repeated origin and loss of adhesive toepads in geckos. PLoS ONE 7:e39429. 10.1371/journal.pone.0039429 PMC3384654 22761794

[msz263-B49] Gao J, Li Q, Wang Z, Zhou Y, Martelli P, Li F, Xiong Z, Wang J, Yang H, Zhang G. 2017 Sequencing, de novo assembling, and annotating the genome of the endangered Chinese crocodile lizard *Shinisaurus crocodilurus*. GigaScience 6(7):1–6. 10.1093/gigascience/gix041 PMC5569961 28595343

[msz263-B50] Gartner GEA, Gamble T, Jaffe AL, Harrison A, Losos JB. 2013 Left-right dewlap asymmetry and phylogeography of *Anolis lineatus* on Aruba and Curaçao. Biol J Linn Soc Lond. 110(2):409–426.

[msz263-B51] Gatesy J, Springer MS. 2014 Phylogenetic analysis at deep timescales: unreliable gene trees, bypassed hidden support, and the coalescence/concatalescence conundrum. Mol Phylogenet Evol. 80:231–266. 25152276 10.1016/j.ympev.2014.08.013

[msz263-B52] Genomic Resources Development Consortium, Arthofer W, Banbury BL, Carneiro M, Cicconardi F, Duda TF, Harris RB, Kang DS, Leaché AD, Nolte V, et al 2015 Genomic resources notes accepted 1 August 2014–30 September 2014. Mol Ecol Resour. 15:228–229. 25424247 10.1111/1755-0998.12340

[msz263-B53] Georges A, Li Q, Lian J, O’Meally D, Deakin J, Wang Z, Zhang P, Fujita M, Patel HR, Holleley CE, et al 2015 High-coverage sequencing and annotated assembly of the genome of the Australian dragon lizard *Pogona vitticeps*. GigaScience 4(1):10. 10.1186/s13742-015-0085-2 PMC4585809 26421146

[msz263-B54] Gilbert C, Meik JM, Dashevsky D, Card DC, Castoe TA, Schaack S. 2014 Endogenous hepadnaviruses, bornaviruses and circoviruses in snakes. Proc Biol Sci. 281(1791):20141122. 25080342 10.1098/rspb.2014.1122 PMC4132678

[msz263-B55] Gowri-Shankar V, Rattray M. 2007 A reversible jump method for Bayesian phylogenetic inference with a nonhomogeneous substitution model. Mol Biol Evol. 24(6):1286–1299. 17347157 10.1093/molbev/msm046

[msz263-B56] Graur D, Li W-H. 2000 Fundamentals of molecular evolution. 2nd ed Sanderland: Sinauer Associates, Inc.

[msz263-B57] Graybeal A. 1994 Evaluating the phylogenetic utility of genes: a search for genes informative about deep divergences among vertebrates. Syst Biol. 43(2):174–193.

[msz263-B302] Grismer JL, Schulte II JA, Alexander A, Wagner P, Travers SL, Buehler MD, Welton LJ, Brown RM. 2016. The Eurasian invasion: Phylogenomic data reveal multiple Southeast Asian origins for Indian dragon lizards. *BMC Evol*. Biol.16(43)11. 10.1186/s12862-016-0611-6 PMC4759850 26896057

[msz263-B58] Groth JG, Barrowclough GF. 1999 Basal divergences in birds and the phylogenetic utility of the nuclear RAG-1 gene. Mol Phylogenet Evol. 12(2):115–123. 10381315 10.1006/mpev.1998.0603

[msz263-B59] Gruber KF, Voss RS, Jansa SA. 2007 Base-compositional heterogeneity in the RAG1 locus among didelphid marsupials: implications for phylogenetic inference and the evolution of GC content. Syst Biol. 56(1):83–96. 17366139 10.1080/10635150601182939

[msz263-B60] Hahn MW, Nakhleh L. 2016 Irrational exuberance for resolved species trees. Evolution 70(1):7–17. 26639662 10.1111/evo.12832

[msz263-B61] Han KL, Braun EL, Kimball RT, Reddy S, Bowie RCK, Braun MJ, Chojnowski JL, Hackett SJ, Harshman J, Huddleston CJ, et al 2011 Are transposable element insertions homoplasy free? An examination using the avian tree of life. Syst Biol. 60(3):375–386. 21303823 10.1093/sysbio/syq100

[msz263-B62] Harmston N, Barešić A, Lenhard B. 2013 The mystery of extreme non-coding conservation. Philos Trans R Soc B. 368(1632):20130021. 10.1098/rstb.2013.0021 PMC3826495 24218634

[msz263-B63] Harrington RC, Near TJ. 2012 Phylogenetic and coalescent strategies of species delimitation in snubnose darters (Percidae: *Etheostoma*). Syst Biol. 61(1):63–79. 21828082 10.1093/sysbio/syr077

[msz263-B64] Hedges SB, Marin J, Suleski M, Paymer M, Kumar S. 2015 Tree of life reveals clock-like speciation and diversification. Mol Biol Evol. 32(4):835–845. 25739733 10.1093/molbev/msv037 PMC4379413

[msz263-B65] Heibl C. 2016 PHYLOCH: R language tree plotting tools and interfaces to diverse phylogenetic software packages. Available from: http://www.christophheibl.de/Rpackages.html, last accessed November 24, 2019.

[msz263-B66] Heled J, Drummond AJ. 2010 Bayesian inference of species trees from multilocus data. Mol Biol Evol. 27(3):570–580. 19906793 10.1093/molbev/msp274 PMC2822290

[msz263-B67] Holland BR, Jarvis PD, Sumner JG. 2013 Low-parameter phylogenetic inference under the general markov model. Syst Biol. 62(1):78–92. 22914976 10.1093/sysbio/sys072

[msz263-B68] Hugall AF, Foster R, Lee M. 2007 Calibration choice, rate smoothing, and the pattern of tetrapod diversification according to the long nuclear gene RAG-1. Syst Biol. 56(4):543–563. 17654361 10.1080/10635150701477825

[msz263-B69] Hunt G. 2011 ScalePhylo. Available from: https://gist.github.com/sckott/938313, last accessed November 24, 2019.

[msz263-B70] Ilves KL, López-Fernández H. 2014 A targeted next-generation sequencing toolkit for exon-based cichlid phylogenomics. Mol Ecol Resour. 14(4):802–811. 24410873 10.1111/1755-0998.12222

[msz263-B71] Jackman TR, Larson A, de Queiroz K, Losos JB. 1999 Phylogenetic relationships and tempo of early diversication in *Anolis* lizards. Syst Biol. 48(2):254–285.

[msz263-B72] Jarvis ED, Mirarab S, Aberer AJ, Li B, Houde P, Li C, Ho SYW, Faircloth BC, Nabholz B, Howard JT, et al 2014 Whole-genome analyses resolve early branches in the tree of life of modern birds. Science 346(6215):1320–1331. 25504713 10.1126/science.1253451 PMC4405904

[msz263-B73] Jeffroy O, Brinkmann H, Delsuc F, Philippe H. 2006 Phylogenomics: the beginning of incongruence? Trends Genet. 22(4):225–231. 16490279 10.1016/j.tig.2006.02.003

[msz263-B74] Jombart T, Dray S. 2016 Adephylo: exploratory analyses for the phylogenetic comparative method. Bioinformatics 26:1–21.

[msz263-B75] Jordan IK, Wolf YI, Koonin EV. 2004 Duplicated genes evolve slower than singletons despite the initial rate increase. BMC Evol Biol. 4(1):22. 15238160 10.1186/1471-2148-4-22 PMC481058

[msz263-B76] Kalyaanamoorthy S, Minh BQ, Wong TKF, Von Haeseler A, Jermiin LS. 2017 ModelFinder: fast model selection for accurate phylogenetic estimates. Nat Methods. 14(6):587. 28481363 10.1038/nmeth.4285 PMC5453245

[msz263-B77] Katoh K, Standley DM. 2013 MAFFT multiple sequence alignment software version 7: improvements in performance and usability. Mol Biol Evol. 30(4):772–780. 23329690 10.1093/molbev/mst010 PMC3603318

[msz263-B78] Katzman S, Kern AD, Bejerano G, Fewell G, Fulton L, Wilson RK, Salama SR, Haussler D. 2007 Human genome ultraconserved elements are ultraselected. Science 317(5840):915. 17702936 10.1126/science.1142430

[msz263-B79] Kearse M, Moir R, Wilson A, Stones-Havas S, Cheung M, Sturrock S, Buxton S, Cooper A, Markowitz S, Duran C, et al 2012 Geneious Basic: an integrated and extendable desktop software platform for the organization and analysis of sequence data. Bioinformatics 28(12):1647–1649. 22543367 10.1093/bioinformatics/bts199 PMC3371832

[msz263-B80] Klopfstein S, Massingham T, Goldman N. 2017 More on the best evolutionary rate for phylogenetic analysis. Syst Biol. 66(5):769–785. 28595363 10.1093/sysbio/syx051 PMC5790136

[msz263-B81] Kolora SRR, Weigert A, Saffari A, Kehr S, Walter Costa MB, Spröer C, Indrischek H, Chintalapati M, Lohse K, Doose G, et al 2019 Divergent evolution in the genomes of closely related lacertids, *Lacerta viridis* and *L. bilineata*, and implications for speciation. Gigascience 8:160. 10.1093/gigascience/giy160 PMC6381762 30535196

[msz263-B82] Lanier HC, Huang H, Knowles LL. 2014 How low can you go? The effects of mutation rate on the accuracy of species-tree estimation. Mol Phylogenet Evol. 70:112–119. 24060367 10.1016/j.ympev.2013.09.006

[msz263-B83] Lanier HC, Knowles LL. 2012 Is recombination a problem for species-tree analyses? Syst Biol. 61(4):691–701. 22215721 10.1093/sysbio/syr128

[msz263-B84] Lavoué S, Sullivan JP, Hopkins CD. 2003 Phylogenetic utility of the first two introns of the S7 ribosomal protein gene in African electric fishes (Mormyroidea: Teleostei) and congruence with other molecular markers. Biol J Linn Soc. 78(2):273–292.

[msz263-B85] Leaché AD, Banbury BL, Linkem CW, De Oca A. 2016 Phylogenomics of a rapid radiation: is chromosomal evolution linked to increased diversification in North American spiny lizards (genus *Sceloporus*)? BMC Evol Biol. 16(1):63. 27000803 10.1186/s12862-016-0628-x PMC4802581

[msz263-B86] Leaché AD, Oaks JR. 2017 The utility of single nucleotide polymorphism (SNP) data in phylogenetics. Annu Rev Ecol Evol Syst. 48(1):69–84.

[msz263-B87] Lemmon AR, Emme SA, Lemmon EM. 2012 Anchored hybrid enrichment for massively high-throughput phylogenomics. Syst Biol. 61(5):727–744. 22605266 10.1093/sysbio/sys049

[msz263-B88] Lenhard B, Sandelin A, Mendoza L, Engström P, Jareborg N, Wasserman WW. 2003 Identification of conserved regulatory elements by comparative genome analysis. J Biol. 2(2):13. 12760745 10.1186/1475-4924-2-13 PMC193685

[msz263-B89] Li C, Hofreiter M, Straube N, Corrigan S, Naylor G. 2013 Capturing protein-coding genes across highly divergent species. Biotechniques 54(6):321–326. 23758502 10.2144/000114039

[msz263-B90] Li H, Handsaker B, Wysoker A, Fennell T, Ruan J, Homer N, Marth G, Abecasis G, Durbin R. 2009 The Sequence Alignment/Map format and SAMtools. Bioinformatics 25(16):2078–2079. 19505943 10.1093/bioinformatics/btp352 PMC2723002

[msz263-B91] Li JN, He C, Guo P, Zhang P, Liang D. 2017 A workflow of massive identification and application of intron markers using snakes as a model. Ecol Evol. 7(23):10042–10055. 29238535 10.1002/ece3.3525 PMC5723593

[msz263-B92] Lind AL, Lai YYY, Mostovoy Y, Holloway AK, Iannucci A, Mak ACY, Fondi M, Orlandini V, Eckalbar WL, Milan M, et al. 2019. Genome of the Komodo dragon reveals adaptations in the cardiovascular and chemosensory systems of monitor lizards. *Nat Ecol Evol* 3:1241–1252. 10.1038/s41559-019-0945-8 PMC6668926 31358948

[msz263-B93] Liu L, Yu L, Edwards SV. 2010 A maximum pseudo-likelihood approach for estimating species trees under the coalescent model. BMC Evol Biol. 10(1):302. 20937096 10.1186/1471-2148-10-302 PMC2976751

[msz263-B94] Liu Y, Zhou Q, Wang Y, Luo L, Yang J, Yang L, Liu M, Li Y, Qian T, Zheng Y, et al 2015 *Gekko japonicus* genome reveals evolution of adhesive toe pads and tail regeneration. Nat Commun. 6(1):11. 10.1038/ncomms10033 PMC4673495 26598231

[msz263-B95] Lockhart PJ, Steel MA, Hendy MD, Penny D. 1994 Recovering evolutionary trees under a more realistic model of sequence evolution. Mol Biol Evol. 11:605–612. 19391266 10.1093/oxfordjournals.molbev.a040136

[msz263-B96] López-Giráldez F, Townsend JP. 2011 PhyDesign: an online application for profiling phylogenetic informativeness. BMC Evol Biol. 11(1):152. 21627831 10.1186/1471-2148-11-152 PMC3124428

[msz263-B97] Luan PT, Ryder OA, Davis H, Zhang YP, Yu L. 2013 Incorporating indels as phylogenetic characters: impact for interfamilial relationships within Arctoidea (Mammalia: Carnivora). Mol Phylogenet Evol. 66(3):748–756. 23147269 10.1016/j.ympev.2012.10.023

[msz263-B98] Maddison WP. 1997 Gene trees in species trees. Syst Biol. 46(3):523–536.

[msz263-B99] Marais G. 2003 Biased gene conversion: implications for genome and sex evolution. Trends Genet. 19(6):330–338. 12801726 10.1016/S0168-9525(03)00116-1

[msz263-B100] McCormack JE, Harvey MG, Faircloth BC, Crawford NG, Glenn TC, Brumfield RT. 2013 A phylogeny of birds based on over 1,500 loci collected by target enrichment and high-throughput sequencing. PLoS One 8(1):e54848. 23382987 10.1371/journal.pone.0054848 PMC3558522

[msz263-B101] McDonald JH, Kreitman M. 1991 Adaptive protein evolution at the Adh locus in *Drosophila*. Nature 351(6328):652–654. 1904993 10.1038/351652a0

[msz263-B102] Miller MA, Pfeiffer W, Schwartz T. 2010. Creating the CIPRES Science Gateway for inference of large phylogenetic trees. In: Proceedings of the Gateway Computing Environments Workshop (GCE); 2010 Nov 14. New Orleans (LA): IEEE. p. 1–8.

[msz263-B103] Minh BQ, Hahn M, Lanfear R. 2018 New methods to calculate concordance factors for phylogenomic datasets. *bioRxiv*. 10.1093/molbev/msaa106 PMC7475031 32365179

[msz263-B104] Mirarab S, Bayzid SM, Boussau B, Warnow T. 2014 Statistical binning enables an accurate coalescent-based estimation of the avian tree. Science 346(6215):1250463. 25504728 10.1126/science.1250463

[msz263-B105] Mirarab S, Reaz R, Bayzid MS, Zimmermann T, Swenson MS, Warnow T. 2014 ASTRAL: genome-scale coalescent-based species tree estimation. Bioinformatics 30(17):i541–i548. 25161245 10.1093/bioinformatics/btu462 PMC4147915

[msz263-B106] Mirarab S, Warnow T. 2015 ASTRAL-II: coalescent-based species tree estimation with many hundreds of taxa and thousands of genes. Bioinformatics 31(12):i44–i52. 26072508 10.1093/bioinformatics/btv234 PMC4765870

[msz263-B107] Moynahan ME, Chiu JW, Koller BH, Jasint M. 1999 BRCA1 controls homology-directed DNA repair. *Mol Cell* 4:511–518. 10.1016/s1097-2765(00)80202-6 10549283

[msz263-B108] Moynahan ME, Pierce AJ, Jasin M. 2001 BRCA2 is required for homology-directed repair of chromosomal breaks. Mol Cell. 7:263–272. 10.1016/s1097-2765(01)00174-5 11239455

[msz263-B109] Nguyen LT, Schmidt HA, Von Haeseler A, Minh BQ. 2015 IQ-TREE: a fast and effective stochastic algorithm for estimating maximum-likelihood phylogenies. Mol Biol Evol. 32(1):268–274. 25371430 10.1093/molbev/msu300 PMC4271533

[msz263-B110] Ni JZ, Grate L, Donohue JP, Preston C, Nobida N, O’Brien G, Shiue L, Clark TA, Blume JE, Ares M. 2007 Ultraconserved elements are associated with homeostatic control of splicing regulators by alternative splicing and nonsense-mediated decay. Genes Dev. 21(6):708–718. 17369403 10.1101/gad.1525507 PMC1820944

[msz263-B111] Nikolaev S, Montoya-Burgos JI, Margulies EH, Bouffard GG, Idol JR, Maduro VVB, Blakesley RW, Guan X, Hansen NF, Maskeri B. 2007 Early history of mammals is elucidated with the ENCODE multiple species sequencing data. PLoS Genet. 3(1):e2. 17206863 10.1371/journal.pgen.0030002 PMC1761045

[msz263-B112] Ogilvie HA, Bouckaert RR, Drummond AJ. 2017 StarBEAST2 brings faster species tree inference and accurate estimates of substitution rates. Mol Biol Evol. 34(8):2101–2114. 28431121 10.1093/molbev/msx126 PMC5850801

[msz263-B113] Ogilvie HA, Heled J, Xie D, Drummond AJ. 2016 Computational performance and statistical accuracy of *BEAST and comparisons with other methods. Syst Biol. 65(3):381–396. 26821913 10.1093/sysbio/syv118 PMC4851174

[msz263-B114] Pál C, Papp B, Hurst LD. 2001 Highly expressed genes in yeast evolve slowly. Genetics 158(2):927–931. 11430355 10.1093/genetics/158.2.927 PMC1461684

[msz263-B115] Paradis E, Claude J, Strimmer K. 2004 APE: analyses of phylogenetics and evolution in R language. Bioinformatics 20(2):289–290. 14734327 10.1093/bioinformatics/btg412

[msz263-B116] Parks M, Cronn R, Liston A. 2012 Separating the wheat from the chaff: mitigating the effects of noise in a plastome phylogenomic data set from *Pinus* L. (Pinaceae). BMC Evol Biol. 12(1):100. 22731878 10.1186/1471-2148-12-100 PMC3475122

[msz263-B117] Perry B, Card DC, McGlothlin JW, Pasquesi GIM, Adams RH, Schield DR, Hales NR, Corbin AB, Demuth JP, Hoffmann FG, et al 2018 Molecular adaptations for sensing and securing prey, and insight into amniote genome diversity, from the garter snake genome. Genome Biol Evol. 10(8):2110–2129. 30060036 10.1093/gbe/evy157 PMC6110522

[msz263-B118] Philippe H, Brinkmann H, Lavrov DV, Littlewood DTJ, Manuel M, Wörheide G, Baurain D. 2011 Resolving difficult phylogenetic questions: why more sequences are not enough. PLoS Biol. 9(3):e1000602. 21423652 10.1371/journal.pbio.1000602 PMC3057953

[msz263-B119] Portik DM, Bauer AM, Jackman TR. 2011 Bridging the gap: western rock skinks (*Trachylepis sulcata*) have a short history in South Africa. Mol Ecol. 20(8):1744–1758. 21371148 10.1111/j.1365-294X.2011.05047.x

[msz263-B120] Portik DM, Smith LL, Bi K. 2016 An evaluation of transcriptome-based exon capture for frog phylogenomics across multiple scales of divergence (Class: Amphibia, Order: Anura). Mol Ecol Resour. 16(5):1069–1083. 27241806 10.1111/1755-0998.12541

[msz263-B121] Posada D. 2016 Phylogenomics for systematic biology. Syst Biol. 65(3):353–356. 27129844 10.1093/sysbio/syw027

[msz263-B122] Posada D, Crandall KA. 2002 The effect of recombination on the accuracy of phylogeny estimation. J Mol Evol. 54(3):396–402. 11847565 10.1007/s00239-001-0034-9

[msz263-B123] Prum RO, Berv JS, Dornburg A, Field DJ, Townsend JP, Lemmon EM, Lemmon AR. 2015 A comprehensive phylogeny of birds (Aves) using targeted next-generation DNA sequencing. Nature 526(7574):569–573. 26444237 10.1038/nature15697

[msz263-B124] Prychitko TM, Moore WS. 1997 The utility of DNA sequences of an intron from the b-Fibrinogen gene in phylogenetic analysis of woodpeckers (Aves: Picidae). Mol Phylogenet Evol. 8(2):193–204. 9299224 10.1006/mpev.1997.0420

[msz263-B125] Pyron RA. 2011 Divergence time estimation using fossils as terminal taxa and the origins of lissamphibia. Syst Biol. 60(4):466–481. 21540408 10.1093/sysbio/syr047

[msz263-B126] Pyron RA, Burbrink FT, Wiens JJ. 2013 A phylogeny and revised classification of Squamata, including 4161 species of lizards and snakes. BMC Evol Biol. 13(1):93. 23627680 10.1186/1471-2148-13-93 PMC3682911

[msz263-B127] Pyron RA, Hendry CR, Chou VM, Lemmon EM, Lemmon AR, Burbrink FT. 2014 Effectiveness of phylogenomic data and coalescent species-tree methods for resolving difficult nodes in the phylogeny of advanced snakes (Serpentes: Caenophidia). Mol Phylogenet Evol. 81:221–231. 25193610 10.1016/j.ympev.2014.08.023

[msz263-B129] Rambaut A, Drummond AJ, Xie D, Baele G, Suchard MA. 2018 Posterior summarisation in Bayesian phylogenetics using Tracer 1.7. Syst Biol. 67(5):901–904. 29718447 10.1093/sysbio/syy032 PMC6101584

[msz263-B130] Ray DA. 2006 SINEs of progress: mobile element applications to molecular ecology. Mol Ecol. 16(1):19–33. 10.1111/j.1365-294X.2006.03104.x 17181718

[msz263-B301] R Core Team. 2016. R: A language and environment for statistical computing. Vienna (Austria): R Foundation for Statistical Computing.

[msz263-B131] Reddy S, Kimball RT, Pandey A, Hosner PA, Braun MJ, Hackett SJ, Han KL, Harshman J, Huddleston CJ, Kingston S, et al 2017 Why do phylogenomic data sets yield conflicting trees? Data type influences the avian tree of life more than taxon sampling. Syst Biol. 66(5):857–879. 28369655 10.1093/sysbio/syx041

[msz263-B132] Roch S, Warnow T. 2015 On the robustness to gene tree estimation error (or lack thereof) of coalescent-based species tree methods. Syst Biol. 64(4):663–676. 25813358 10.1093/sysbio/syv016

[msz263-B133] Roscito JG, Sameith K, Pippel M, Francoijs KJ, Winkler S, Dahl A, Papoutsoglou G, Myers G, Hiller M. 2018 The genome of the tegu lizard *Salvator merianae*: combining Illumina, PacBio, and optical mapping data to generate a highly contiguous assembly. GigaScience 7:giy141. 10.1093/gigascience/giy141 PMC6304105 30481296

[msz263-B134] Ruane S, Raxworthy CJ, Lemmon AR, Lemmon EM, Burbrink FT. 2015 Comparing species tree estimation with large anchored phylogenomic and small Sanger-sequenced molecular datasets: an empirical study on Malagasy pseudoxyrhophiine snakes. BMC Evol Biol. 15(1):221. 26459325 10.1186/s12862-015-0503-1 PMC4603904

[msz263-B135] Salichos L, Rokas A. 2013 Inferring ancient divergences requires genes with strong phylogenetic signals. Nature 497(7449):327–331. 23657258 10.1038/nature12130

[msz263-B136] Schliep KP. 2011 phangorn: phylogenetic analysis in R. Bioinformatics 27(4):592–593. 21169378 10.1093/bioinformatics/btq706 PMC3035803

[msz263-B137] Shaw J, Lickey EB, Schilling EE, Small RL. 2007 Comparison of whole chloroplast genome sequences to choose noncoding regions for phylogenetic studies in angiosperms: the tortoise and the hare III. Am J Bot. 94(3):275–288. 21636401 10.3732/ajb.94.3.275

[msz263-B138] Shen XX, Hittinger CT, Rokas A. 2017 Contentious relationships in phylogenomic studies can be driven by a handful of genes. Nat Ecol Evol. 1(5):1–10. 28812701 10.1038/s41559-017-0126 PMC5560076

[msz263-B139] Shen XX, Salichos L, Rokas A. 2016 A genome-scale investigation of how sequence, function, and tree-based gene properties influence phylogenetic inference. Genome Biol Evol. 8(8):2565–2580. 27492233 10.1093/gbe/evw179 PMC5010910

[msz263-B140] Simmons MP, Ochoterena H. 2000 Gaps as characters in sequence-based phylogenetic analyses. Syst Biol. 49(2):369–381. 12118412

[msz263-B141] Singhal S, Grundler M, Colli G, Rabosky DL. 2017 Squamate Conserved Loci (SqCL): a unified set of conserved loci for phylogenomics and population genetics of squamate reptiles. Mol Ecol Resour. 17(6):e12–e24. 28417603 10.1111/1755-0998.12681

[msz263-B142] Song B, Cheng S, Sun Y, Zhong X, Jin J, Guan R, Murphy RW, Che J, Zhang Y, Liu X. 2015 A genome draft of the legless anguid lizard, *Ophisaurus gracilis*. GigaScience 4(1):3. 25859342 10.1186/s13742-015-0056-7 PMC4391233

[msz263-B143] Springer MS, Gatesy J. 2016 The gene tree delusion. Mol Phylogenet Evol. 94:1–33. 26238460 10.1016/j.ympev.2015.07.018

[msz263-B144] Stamatakis A. 2014 RAxML version 8: a tool for phylogenetic analysis and post-analysis of large phylogenies. Bioinformatics 30(9):1312–1313. 24451623 10.1093/bioinformatics/btu033 PMC3998144

[msz263-B145] Stamatakis A. 2015 The RAxML v8.2.X manual. Heidelb Inst Theor Stud. 1–61.

[msz263-B146] Streicher JW, Wiens JJ. 2017 Phylogenomic analyses of more than 4000 nuclear loci resolve the origin of snakes among lizard families. Biol Lett. 13(9):20170393. 28904179 10.1098/rsbl.2017.0393 PMC5627172

[msz263-B147] Tarver JE, Sperling EA, Nailor A, Heimberg AM, Robinson JM, King BL, Pisani D, Donoghue PCJ, Peterson KJ. 2013 miRNAs: small genes with big potential in metazoan phylogenetics. Mol Biol Evol. 30(11):2369–2382. 23913097 10.1093/molbev/mst133

[msz263-B148] Townsend JP. 2007 Profiling phylogenetic informativeness. Syst Biol. 56(2):222–231. 17464879 10.1080/10635150701311362

[msz263-B149] Townsend JP, Su Z, Tekle YI. 2012 Phylogenetic signal and noise: predicting the power of a data set to resolve phylogeny. Syst Biol. 61(5):835–849. 22389443 10.1093/sysbio/sys036

[msz263-B150] Townsend TM, Larson A, Louis E, Macey JR. 2004 Molecular phylogenetics of squamata: the position of snakes, amphisbaenians, and dibamids, and the root of the squamate tree. Syst Biol. 53(5):735–757. 15545252 10.1080/10635150490522340

[msz263-B151] Tucker DB, Colli GR, Giugliano LG, Hedges SB, Hendry CR, Lemmon EM, Lemmon AR, Sites JW, Pyron RA. 2016 Methodological congruence in phylogenomic analyses with morphological support for teiid lizards (Sauria: Teiidae). Mol Phylogenet Evol. 103:75–84. 27395779 10.1016/j.ympev.2016.07.002

[msz263-B152] Ullate-Agote A, Milinkovitch MC, Tzika AC. 2014 The genome sequence of the corn snake (*Pantherophis guttatus*), a valuable resource for EvoDevo studies in squamates. Int J Dev Biol. 58(10-11-12):881–888. 26154328 10.1387/ijdb.150060at

[msz263-B153] Vicoso B, Emerson JJ, Zektser Y, Mahajan S, Bachtrog D. 2013 Comparative sex chromosome genomics in snakes: differentiation, evolutionary strata, and lack of global dosage compensation. PLoS Biol. 11(8):e1001643. 24015111 10.1371/journal.pbio.1001643 PMC3754893

[msz263-B154] Vonk FJ, Casewell NR, Henkel CV, Heimberg AM, Jansen HJ, McCleary RJR, Kerkkamp HME, Vos RA, Guerreiro I, Calvete JJ, et al 2013 The king cobra genome reveals dynamic gene evolution and adaptation in the snake venom system. Proc Natl Acad Sci U S A. 110(51):20651–20656. 24297900 10.1073/pnas.1314702110 PMC3870661

[msz263-B155] Warnefors M, Hartmann B, Thomsen S, Alonso CR. 2016 Combinatorial gene regulatory functions underlie ultraconserved elements in *Drosophila*. Mol Biol Evol. 33(9):2294–2306. 27247329 10.1093/molbev/msw101 PMC4989106

[msz263-B156] Whelan S, Goldman N. 2001 A general empirical model of protein evolution derived from multiple protein families using a maximum-likelihood approach. Mol Biol Evol. 18(5):691–699. 11319253 10.1093/oxfordjournals.molbev.a003851

[msz263-B157] Wickett NJ, Mirarab S, Nguyen N, Warnow T, Carpenter E, Matasci N, Ayyampalayam S, Barker MS, Burleigh JG, Gitzendanner MA, et al 2014 Phylotranscriptomic analysis of the origin and early diversification of land plants. Proc Natl Acad Sci U S A. 111(45):E4859–E4868. 25355905 10.1073/pnas.1323926111 PMC4234587

[msz263-B158] Wiens JJ, Hutter CR, Mulcahy DG, Noonan BP, Townsend TM, Sites JW, Reeder TW. 2012 Resolving the phylogeny of lizards and snakes (Squamata) with extensive sampling of genes and species. Biol Lett. 8(6):1043–1046. 22993238 10.1098/rsbl.2012.0703 PMC3497141

[msz263-B159] Woolfe A, Goodson M, Goode DK, Snell P, McEwen GK, Vavouri T, Smith SF, North P, Callaway H, Kelly K, et al 2004 Highly conserved non-coding sequences are associated with vertebrate development. PLoS Biol. 3(1):e7. 15630479 10.1371/journal.pbio.0030007 PMC526512

[msz263-B160] Xiong Z, Li F, Li Q, Zhou L, Gamble T, Zheng J, Kui L, Li C, Li S, Yang H, et al 2016 Draft genome of the leopard gecko, *Eublepharis macularius*. GigaScience 5(1):6. 27784328 10.1186/s13742-016-0151-4 PMC5080775

[msz263-B161] Yang Z. 1998 On the best evolutionary rate for phylogenetic analysis. Syst Biol. 47(1):125–133. 12064232 10.1080/106351598261067

[msz263-B162] Yang Z. 2007 PAML 4: phylogenetic analysis by maximum likelihood. Mol Biol Evol. 24(8):1586–1591. 17483113 10.1093/molbev/msm088

[msz263-B163] Yang Z, Nielsen R. 2002 Codon-substitution models for detecting molecular adaptation at individual sites along specific lineages. Mol Biol Evol. 19(6):908–917. 12032247 10.1093/oxfordjournals.molbev.a004148

[msz263-B164] Yin W, Wang Z-J, Li Q-y, Lian J-M, Zhou Y, Lu B-Z, Jin L-J, Qiu P-X, Zhang P, Zhu W-B, et al 2016 Evolutionary trajectories of snake genes and genomes revealed by comparative analyses of five-pacer viper. Nat Commun. 7(1):13107. 27708285 10.1038/ncomms13107 PMC5059746

[msz263-B165] Yurchenko AA, Recknagel H, Elmer KR. 2019 Chromosome-level assembly of the common lizard (*Zootoca vivipara*) genome. bioRxiv: 520528. 10.1093/gbe/evaa161 PMC7643610 32835354

[msz263-B166] Zhang C, Rabiee M, Sayyari E, Mirarab S. 2018 ASTRAL-III: polynomial time species tree reconstruction from partially resolved gene trees. BMC Bioinformatics 19(S6):153. 29745866 10.1186/s12859-018-2129-y PMC5998893

[msz263-B167] Zhang L, Li WH. 2004 Mammalian housekeeping genes evolve more slowly than tissue-specific genes. Mol Biol Evol. 21(2):236–239. 14595094 10.1093/molbev/msh010

[msz263-B168] Zheng Y, Wiens JJ. 2016 Combining phylogenomic and supermatrix approaches, and a time-calibrated phylogeny for squamate reptiles (lizards and snakes) based on 52 genes and 4162 species. Mol Phylogenet Evol. 94:537–547. 26475614 10.1016/j.ympev.2015.10.009

[msz263-B169] Zhu Q. 2014 BeforePhylo version 0.9.0. Available from: https://github.com/qiyunzhu/BeforePhylo, last accessed November 24, 2019.

