# Supplementary figures and images for: Optimizing Phylogenomics with Rapidly Evolving Long Exons: Comparison with Anchored Hybrid Enrichment and Ultraconserved Elements
Source: Mol Biol Evol. 2019 Nov 9;37(3):904–22. doi: 10.1093/molbev/msz263 (PMC7038749; doi:10.1093/molbev/msz263)

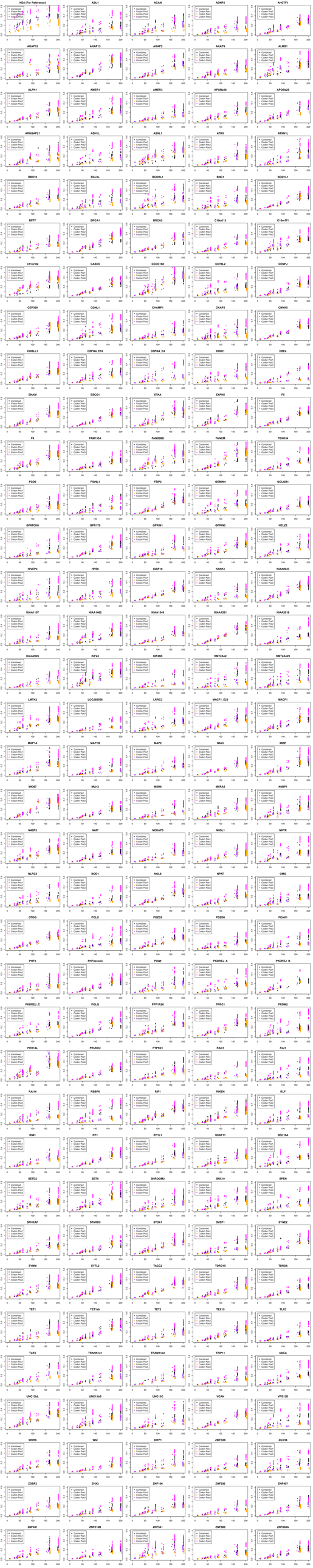

Supplement: msz263_Supplementary_Data [file msz263_supplementary_data.zip › FigS4.pdf]
